# Development of high-throughput SNP-based genotyping in *Acacia auriculiformis* x *A*. *mangium* hybrids using short-read transcriptome data

**DOI:** 10.1186/1471-2164-13-726

**Published:** 2012-12-24

**Authors:** Melissa ML Wong, Charles H Cannon, Ratnam Wickneswari

**Affiliations:** 1 School of Environmental and Natural Resource Sciences, Faculty of Science and Technology, Universiti Kebangsaan Malaysia, UKM Bangi 43600, Selangor, Malaysia; 2 Ecological Evolution Group, Xishuangbanna Tropical Botanical Garden, Chinese Academy of Science, Menglun, Mengla, Yunnan, 666303, P. R. China; 3 Department of Biological Sciences, Texas Tech University, Lubbock, TX, 79409, USA

## Abstract

**Background:**

Next Generation Sequencing has provided comprehensive, affordable and high-throughput DNA sequences for Single Nucleotide Polymorphism (SNP) discovery in *Acacia auriculiformis* and *Acacia mangium*. Like other non-model species, SNP detection and genotyping in *Acacia* are challenging due to lack of genome sequences. The main objective of this study is to develop the first high-throughput SNP genotyping assay for linkage map construction of *A*. *auriculiformis* x *A*. *mangium* hybrids.

**Results:**

We identified a total of 37,786 putative SNPs by aligning short read transcriptome data from four parents of two *Acacia* hybrid mapping populations using Bowtie against 7,839 *de novo* transcriptome contigs. Given a set of 10 validated SNPs from two lignin genes, our *in silico* SNP detection approach is highly accurate (100%) compared to the traditional *in vitro* approach (44%). Further validation of 96 SNPs using Illumina GoldenGate Assay gave an overall assay success rate of 89.6% and conversion rate of 37.5%. We explored possible factors lowering assay success rate by predicting exon-intron boundaries and paralogous genes of *Acacia* contigs using *Medicago truncatula* genome as reference. This assessment revealed that presence of exon-intron boundary is the main cause (50%) of assay failure. Subsequent SNPs filtering and improved assay design resulted in assay success and conversion rate of 92.4% and 57.4%, respectively based on 768 SNPs genotyping. Analysis of clustering patterns revealed that 27.6% of the assays were not reproducible and flanking sequence might play a role in determining cluster compression. In addition, we identified a total of 258 and 319 polymorphic SNPs in *A*. *auriculiformis* and *A*. *mangium* natural germplasms, respectively.

**Conclusion:**

We have successfully discovered a large number of SNP markers in *A*. *auriculiformis* x *A*. *mangium* hybrids using next generation transcriptome sequencing. By using a reference genome from the most closely related species, we converted most SNPs to successful assays. We also demonstrated that Illumina GoldenGate genotyping together with manual clustering can provide high quality genotypes for a non-model species like *Acacia*. These SNPs markers are not only important for linkage map construction, but will be very useful for hybrid discrimination and genetic diversity assessment of natural germplasms in the future.

## Background

The *Acacia auriculiformis* x *A*. *mangium* hybrid is emerging as an important forest tree for pulpwood production in South East Asia. Marker-assisted breeding is a promising approach for selection of superior trees with improved wood and pulp properties for the establishment of forest plantations. Previous efforts to develop molecular markers such as Cleaved Amplified Polymorphic Sequence (CAPS) [1], genomic - Simple Sequence Repeat (SSR) [2] and Expressed Sequence Tag - Simple Sequence Repeat (EST-SSR) [3] for *Acacia* hybrid did not generate sufficient markers for linkage map construction because they were either monomorphic or not fully informative for the biparental mapping populations. Development of molecular marker from narrow genetic background such as the parents of the mapping population is an effective way to generate informative markers for linkage mapping. Towards this end, Single Nucleotide Polymorphism (SNP) is the ideal marker because it provides affordable and high-throughput genotyping compared to other markers [4]. A SNP is a single base change that occurs in at least 1% of the population [5]. SNPs are also co-dominant, bi-allelic, abundant in the genome [6] and thus, suitable for low genetic diversity species such as *A*. *mangium*[7]. Besides linkage map construction, SNPs can be used in genetic diversity assessment of natural germplasms, estimation of outcrossing rate in natural germplasms and seed orchards, and more importantly, clone and hybrid identification in breeding program of both species. To date, only one study reported the genetic diversity of *A*. *mangium* involving the use of Restriction Fragment Length Polymorphism (RFLP) markers [8]. Genetic diversity of *A*. *auriculiformis* has been studied using isozyme markers [9,10] and Sequence Characterized Amplified Region (SCAR) [11]. Although there is no study that compare the genetic diversity of both species, the lower SNP frequency observed in the transcriptome of *A*. *mangium*[12] suggested that *A*. *mangium* has lower genetic diversity than *A*. *auriculiformis*.

There are two main strategies to develop SNP markers, namely *in vitro* and *in silico* method. The *in vitro* method involves polymorphism screening using DNA sequencing while *in silico* method detects polymorphisms in DNA sequences of different individuals using computer sequence analysis [13]. Although *in silico* method is cheaper and less labour intensive compared to *in vitro* detection, it is more prone to sequencing errors and low sequence coverage [14]. Currently, Next Generation Sequencing (NGS) offers affordable, high-throughput, and accurate sequence data generation. NGS has been proven highly effective for *in silico* SNP detection in many plants with reference genome such as Arabidopsis [15]. For non-model species, several approaches have been adopted to overcome the lack of reference genome: a) generation of genome sequences using the Reduced Representation Library (RRL) method [16-18]; b) use of reference genome from closely related species such as catfish [19]; c) Use of gene index as reference [20]; d) *de novo* transcriptome sequencing and assembly [12,21-23]. Many reported studies on *de novo* transcriptome sequencing utilized 454 sequences that give longer read length for SNPs discovery (e.g., *Eucalyptus grandis*[21] and maize [22]). Although various methods have been reported, it is difficult to apply the results of these findings to obtain similar results due to the differences in read quality, sequence coverage and preference of mapping and SNPs calling tools. It is important to understand the limitations and error rate of each dataset for effective *in silico* SNP detection [24].

Medium- to high-throughput custom SNP genotyping technologies such as Illumina GoldenGate, KBiosciences KASPar and Sequenom iPlex, which differ in assay type, throughput, multiplexing and cost are suitable for linkage map construction [25]. Among these technologies, the Illumina GoldenGate Assay has been widely applied in many plant species [26]. It has been demonstrated in human genome to provide affordable genotyping which has high reliability, reproducibility and multiplexing of up to 1,536 SNPs [27]. In Illumina GoldenGate Assay, three oligonucleotides are designed for each SNP locus using the submitted flanking sequence around the SNP site. A minimum of 50 bp flanking sequence upstream and downstream of the SNP site is required for submission [28]. Two oligos are specific to each allele of the SNP site, called Allele-Specific Oligos (ASO1 and ASO2), while a third oligo known as Locus Specific Oligo (LSO) carries a unique address sequence and hybridizes several bases downstream from the SNP site. The assay involves several steps such as DNA activation, oligonucleotide hybridization, extension and ligation, universal PCR, array hybridization and scanning. For SNP genotyping using Illumina BeadXpress Reader machine, genomic DNA is first biotinylated, attached to the oligonucleotides and bounded to streptavidin-coated paramagnetic beads [29]. The extension and ligation of hybridized oligonucleotides provide PCR template using three universal PCR primers in which two are Cy3 and Cy5 fluorescence-labelled. The non-fluorescent strand of PCR product is removed through its 5’ biotin group to generate single-stranded DNA for hybridization to VeraCode Beads. After hybridization, the BeadXpress Reader machine scanned for fluorescence signals on VeraCode Bead Plate and exports the intensity values to GenCall software. GenCall software uses a clustering algorithm known as GenTrain and calculates a quality score for each genotype [30]. The intensity values for each of the two-color channels, commonly referred to as A and B, are normalized and plotted to display distinct patterns or clusters to represent AA, AB and BB signal profiles. The AA, AB and BB clusters correspond to homozygous genotype for allele A, heterozygous genotype and homozygous genotype for allele B, respectively.

The development of high-throughput SNP assay in *Acacia* can be challenging for several reasons. Without a reference genome, several factors are known to affect the success of a SNP assay such as the presence of exon-intron boundaries, secondary SNPs and indels in the flanking region, paralogous genes, genome complexity and repetitive sequence. The assay success rate has been reportedly lower in conifer forest species with complex genome (e.g., 67% in *Pinus radiata*[31] and *Pinus pinaster*[32], and 82% in spruces [33]). This factor may not be an issue for tropical hardwoods, which typically have smaller genomes and considerably less repetitive sequences than conifers. Although SNP transferability to other species has been reported in several plant species [34,35], SNP development in interspecific crosses has only been reported for a few forest and aquaculture species that remained largely “wild” and naturally outcrossing [19,35,36]. When using interspecific crosses, genomic similarity between species must be high to allow amplification and hybridization in SNP genotyping. The genes of *A*. *auriculiformis* and *A*. *mangium* have been reported to share 99% similarity in nucleotide level [12], and thus sequence similarity is not a concern. The overall success of SNPs development in *Acacia* hybrid will depend on short read sequence quality, a highly robust SNP detection approach to identify sequencing error and with enough sensitivity to detect rare SNPs to increase assay successful rate, appropriate assay design and genotype calling approach to obtain high quality genotypes.

In this study, we aimed to develop high-throughput SNP genotyping assay for *A*. *auriculiformis* x *A*.*mangium* hybrid with the ultimate objective of linkage map construction. We sequenced the transcriptomes of the parents of two mapping populations and mapped the short reads against a set of gene contigs to discover SNP markers. We evaluated our SNP detection approach based on a set of validated SNPs from two lignin genes detected using *in vitro* approach and further validated 96 SNPs using Illumina GoldenGate Assay. We also investigated several factors affecting assay success rate based on 96-plex validation. Based on these findings, we further improved the SNP detection approach and designed Illumina GoldenGate Assay consisting of 768 SNPs. The clustering patterns were analyzed to evaluate the reproducibility of Illumina GoldenGate Assay. In addition, we identified polymorphic SNPs that can be transferred to natural germplasms of *A*. *auriculiformis* and *A*. *mangium*.

## Results

### Transcriptome sequencing and SNP detection

Sequencing of normalized cDNA library from sample AA3 and AM22 produced 4,320,132 and 2,921,811 48 bp paired-end reads, respectively. Removal of SMART adaptors and concatemers resulted in 8,566,801 and 5,170,366 single-end reads for AA3 and AM22, respectively. The read generation and pre-processing for individual AA6 and AM20 were described in Wong *et al*. 2011 [12]. A total of 7,839 contigs with lengths ranging from 200-15,266 bp where 6,771 and 1,068 contigs came from AA6 and AM20 *de novo* transcriptome assemblies, respectively were selected as reference sequences (subsequently known as AArefseq) for SNP detection. Bowtie mapped about 21.15-39.71% filtered reads from each dataset to AArefseq (Table 1) and a total of 37,786 putative SNPs were called by Samtools after excluding redundant SNPs present in at least two inviduals. Generally, BWA mapped about 50% less reads to AArefseq compared to Bowtie resulting in 55-65% fewer putative SNPs being called. Higher proportion of mapped reads from dataset AA3 using both software was observed compared to sample AM22 as a result of better cDNA normalization. All putative SNPs contained scores that ranged from 20 to 228. About 80-87% of the putative SNPs detected by BWA were also detected by Bowtie (data not shown).

**Table 1:** Summary of SNP detection approach

**SNP detection**	**AA6**	**AM20**	**AA3**	**AM22**
**BWA**				
1) Percentage of mapped reads	11.92%	11.31%	15.84%	11.57%
2) Putative SNPs	8,345	4,652	3,885	972
**Bowtie**				
1) Percentage of mapped reads	22.57%	21.15%	39.71%	22.21%
2) Putative SNPs	18,684	11,093	10,993	2,516
3) Number of SNPs after Filter 1	11,439	5,738	6,711	861
4) Number of SNPs after Filter 2	6,199	3,179	4,508	629
5) Number of SNPs after Filter 2 with design score >; 0.4	5,094	2,210	3,996	438
6) Validation rate of SNPs after Filter 2	50%	50%	20%	26.7%

### Comparison of *in vitro* and *in silico* methods

**Figure 1:**
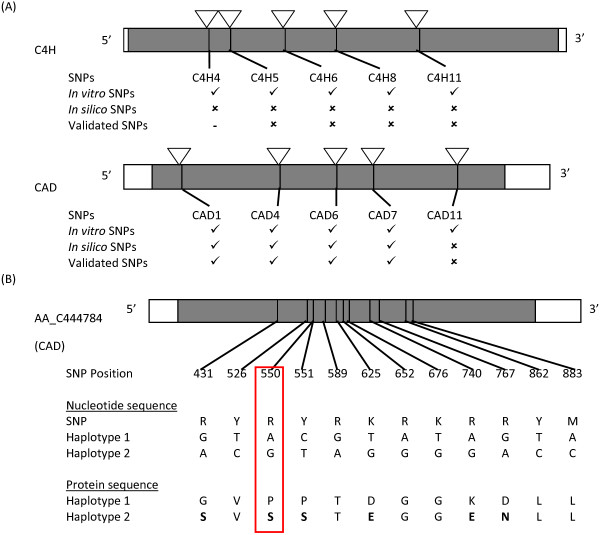
**Comparison of *in vitro *and *in silico *SNP detection approach using validated SNPs from two lignin genes.** (**A**) The SNPs detected *in vitro* and *in silico* approach in C4H (top) and CAD (bottom) gene were compared to SNPs validated using Illumina GoldenGate assay. The tick and cross indicates polymorphic and monomorphic SNP, respectively, while a dash indicates a failed assay; (**B**) The nucleotide and protein sequence of two SNP haplotypes found in CAD gene observed in individual AA3. The haplotypes consist of 12 SNPs within 452 bp. Six non-synonymous SNPs from haplotype 2 which caused a change in amino acid sequences are indicated in bold font. The SNP that is detected only by Bowtie/Samtools approach are circled in red.

To evaluate the accuracy of putative SNPs, we compared the SNPs detected in the coding region of two lignin genes, namely C4H and CAD gene using the current *in silico* approach and *in vitro* approach. We found out that *in silico* method is highly accurate compared to *in vitro* method. A total of 10 validated SNPs, namely 5 SNPs for each gene were compared to the SNPs detected in the present study (Figure 1A). Out of 5 validated SNPs in coding region of C4H gene, only four SNPs were assayed successfully. All four SNPs were monomorphic, as predicted by the *in silico* method. We detected a tri-allelic SNP several bp away from SNP site in the only failed assay of C4H gene, namely C4H4. Out of 5 validated SNPs in CAD gene, four SNPs were polymorphic and one was monomorphic in consistent with the results from *in silico* method. Only 4 out of 5 SNPs predicted by *in vitro* method were polymorphic. Out of 9 successfully assayed and validated SNPs in both genes, the accuracy of *in silico* and *in vitro* method was 100% and 44%, respectively.

When comparing both methods, we observed two SNP haplotypes consisting of 12 SNPs within 452 bp region in the CAD gene sequence of parent AA3 (Figure 1B). Using the *in vitro* method, 3 out of 8 sequenced clones were found to contain haplotype 2. However, haplotype 2 was not noticeable as there are many sequencing errors in the multiple sequence alignment. Our observation that some 50 bp short reads spanned two to three SNPs confirmed the presence of this haplotype. Another important finding was six out of twelve SNPs in haplotype 2 were non-synonymous SNPs, which resulted in a change in 5 amino acid sequences.

To determine the SNPs filtering stringency, we checked the SNP score for the validated SNPs including the SNPs from the haplotype. We found that the validated SNP which contained the lowest SNP score was a SNP at position 550 of contig AA_C444784 (CAD gene) (Figure 1B). This SNP has a SNP score of 52, MAF of 8% and coverage of 191 reads when detected by Bowtie/Samtools. However, this SNP was missed by BWA/Samtools approach because it was not called by Samtools, although most reads were mapped by BWA. Based on these findings, we chose Bowtie as the preferred mapping software and increased the filtering parameters to a minimum SNP score cutoff of 50 and MAF of 8% (Filter 1). After applying Filter 1, a total of 14,515 SNPs from four parents were obtained. After submission to ADT, a total of 12,176 SNPs representing 4,059 contigs had final design score of more than 0.4.

### Validation of *in silico* SNP detection approach

**Figure 2:**
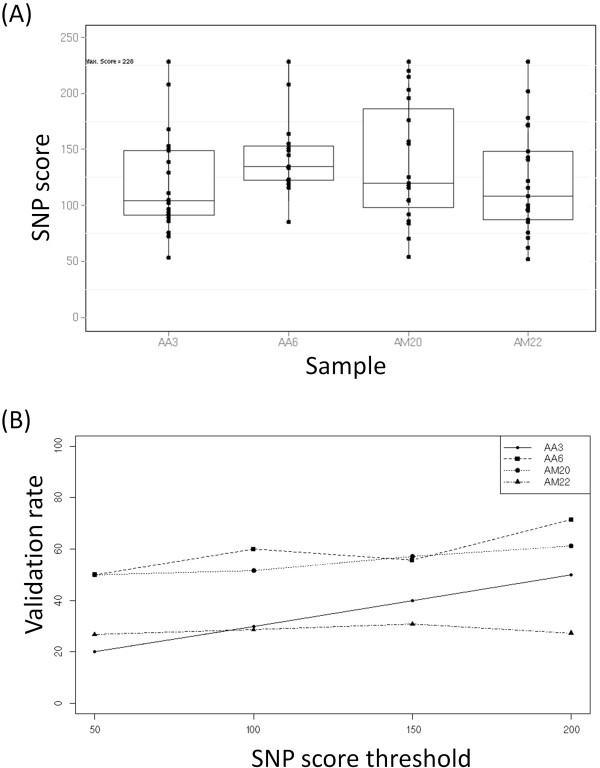
**SNP score and validation rate of 96-plex genotyping for each *Acacia *hybrid parent.** (**A**) Box plot of SNP score distribution for each parent (**B**) Validation rates based on minimum SNP score cutoff for each parent.

A set of 96 SNPs with different SNP scores which ranged from 50 to 228, were selected to validate the improved *in silico* SNP detection approach (Figure 2A). Out of 96 SNPs, 86 assays were successful, giving an assay success rate of 89.6%. Despite a lower concentration of DNA used (20-30 ng/μl) compared to the recommended concentration 50 ng/μl, 99% of the samples had a minimum call rate of 95%. The reproducibility rate of duplicated AM20 samples based on successfully assayed SNPs was 100% after excluding missing data.

An overall conversion rate of 37.5% was obtained. 36 SNPs out of 86 successfully assayed SNPs were polymorphic, where 13 and 6 SNPs were polymorphic in WD and FL population, respectively, while 17 SNPs were polymorphic in both populations. We also observed that 25% of the polymorphic SNPs in WD population can be transferred to FL population. Out of the remaining 50 monomorphic SNPs, 12 SNPs were interspecific SNPs (AA x BB →AB), while 7 SNPs were identified as SNPs from paralogous genes (all genotypes are AB). We found a significant relationship between SNP score and validation rate as expected (p<0.0001) and a correlation of 0.42. Generally, SNP validation rate for each parent increased as the minimum SNP score threshold increased, except individual AM22 (Figure 2B).

Next, we compared the successfully assayed SNPs to the SNPs detected from BWA alignments (Table 2). Since we did not validate a set of SNPs detected only from BWA alignments, we can only compare the accuracy of SNPs detected from both BWA and Bowtie alignments. The validation rates of SNPs detected from both BWA and Bowtie alignments ranged from 33%-77.8%, which were much higher than the validation rate of SNPs detected from Bowtie alignments alone. The highest validation rate was observed in individual AA6, similar to the SNP detection result from Bowtie alignments (20-50%). The sensitivity of BWA/Samtools approach ranged from 62.5% to 80% and the estimated false negative rate ranged from 20% to 37.5%. An average of 28.3% SNPs failed to be detected from BWA alignments despite a higher accuracy in SNPs detected from both BWA and Bowtie alignments.

**Table 2:** **The number of SNPs from 96**-**plex validation detected using BWA**/**Samtools approach**

**Individual**	**No. of successful assays**	**Number of SNPs also detected by BWA**		**Number of SNPs not detected by BWA**		**Sensitivity**	**False negative**
		**True SNPs**	**Total**	**True SNPs**	**Total**		
AA3	25	5 (45.5%)	11	2	14	71.4%	28.6%
AA6	28	14 (77.8%)	18	5	10	66.7%	33.3%
AM20	28	12 (70.6%)	17	3	11	80.0%	20.0%
AM22	26	5 (33.0%)	15	3	11	62.5%	37.5%

### Factors influencing assay failure in Illumina GoldenGate Assay

To determine whether the SNP final design score influences assay failure, we checked the final design score of all failed assays which ranged from 0.418 to 0.998. We found that the design score of failed assays and successful assays were not significantly different using t-test (p≤0.05). Out of 86 working assays, 20 SNPs (23%) had design scores in the range of 0.4 to 0.6. Chisquare test indicated that there is no significant difference (p≤0.05) in assay success rates between SNPs with final design score of 0.4-0.6 and more than 0.6.

**Figure 3:**
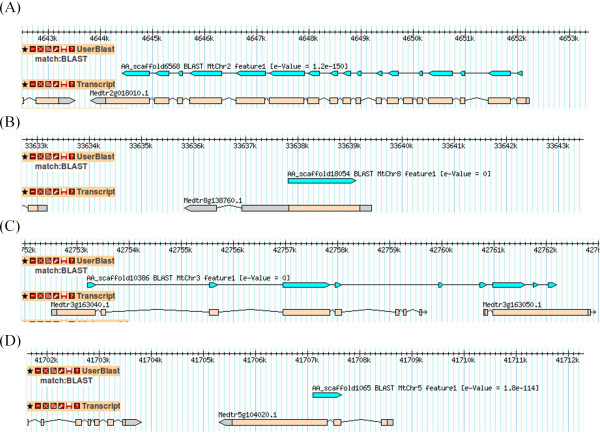
**Exon**-**intron boundaries prediction of *Acacia *contigs using *Medicago truncatula *genome as reference.** The Blastn alignments of *Acacia* contigs to *M*. *truncatula* v2.0 genome using Phytozome v8 showed in Gbrowser consist of four main types: (**A**) Acacia contig that shared highly conserved gene structure with *M*. *truncatula* genes; (**B**) *Acacia* contig that aligned to exonic or untranslated region of *M*. *truncatula* genes; (**C**) *Acacia* contig that spanned two *M*. *truncatula* genes located next to each other in the genome. These two *M*. *truncatula* genes are annotated as a single gene in the *M*. *truncatula* v3.5 genome and thus matching the *Acacia* contig alignment; (**D**) *Acacia* contig that shared less conserved gene structure with *M*. *truncatula* genes.

To investigate how exon-intron boundaries can affect assay failure, we predicted the exon-intron boundaries in the *Acacia* contigs based on homologous *M*. *truncatula* genes. When the contigs were searched against *M*. *truncatula* genome (Mt3.0) using Blastn in Phytozome v8 [37], we observed that the *Acacia* contigs mapped to *M*. *truncatula* genome in four ways: A) *Acacia* contig mapped to highly homologous genes in *M*. *truncatula* genome (Figure 3A); B) *Acacia* contig mapped to two genes that were adjacent to each other in *M*. *truncatula* genome (Figure 3B); C) *Acacia* contig mapped to the intronic region of its homologous gene in *M*. *truncatula* genome (Figure 3C); D) *Acacia* contig mapped to the untranslated region of *M*. *truncatula* gene (Figure 3D). Further analysis of *Acacia* contigs that mapped to *M*. *truncatula* revealed that observations in case B occurred due to the different genome versions used in Phytozome and the present study. In the latest version that Mt3.5 used in the present study, the two *M*. *truncatula* genes were annotated as a single gene, thus matching the *Acacia* contig alignment.

**Figure 4:**
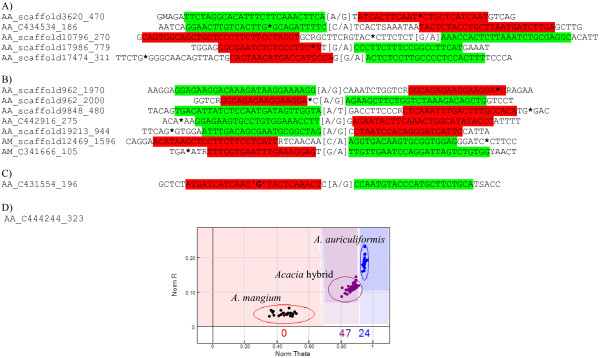
**Effects of exon**-**intron boundary and secondary SNP on assay success rate in 96-plex validation.** (**A**) The flanking regions of failed SNP assays that contain predicted exon-intron boundaries. Three out of five failed assays contained predicted exon-intron boundaries (marked with asterisk) which occur within oligonucloetide binding site; (**B**) The flanking regions of successful assays containing predicted exon-intron boundary (marked with asterisk) which are outside of oligonucloetide binding site in six out of seven assays; (**C**) SNP flanking region of failed assay indicating a secondary SNP (in single quotes) in LSO binding site; (**D**) Clustering profiles of assays containing a potential secondary SNP in flanking region; The two types of oligonucleotides, namely ASO and LSO were highlighted in green and red, respectively.

Using this method, we were able to map 57 out of 85 contigs from 96 validated SNPs to *M*. *truncatula* genome to predict exon-intron boundaries. Out of ten failed assays, we found five SNPs with predicted exon-intron boundaries located at 3-35 bp away from SNP site. However, not all of the SNP assays containing predicted exon-intron boundaries in the flanking region were failed assays. Out of 86 successful assays, there are seven SNPs contained predicted exon-intron boundaries that were 10–33 bp away from SNP. The presence of predicted exon-intron boundaries and distance of exon-intron boundaries from SNP site did not seem to determine assay failure. To identify the difference between these two groups of assays carrying predicted exon-intron boundaries, we analyzed the flanking region of all 12 SNPs with predicted exon-intron boundaries. By mapping the oligonucleotide sequences (ASO1, ASO2 and LSO) obtained from the OPA manifest file to the SNP flanking region, we found that the oligonucleotide binding sites were usually located about 19–29 bp upstream and downstream of the SNP. Out of five failed SNPs, the predicted exon-intron boundaries of three SNPs, namely AA_scaffold3620_470, AA_C434534_186 and AA_scaffold17986_779 were located on the oligonucleotide binding sites while the rest contained predicted exon-intron boundaries outside the oligonucleotide binding sites (Figure 4A). In the seven successfully assayed SNPs, six SNPs contained the predicted exon-intron boundary outside oligonucleotide binding sites while the predicted exon-intron boundaries of one SNP fell between the last and second last nucleotide of LSO binding site (Figure 4B). Generally, predicted exon-intron boundary was located within oligonucleotide binding sites in the majority of failed assays and located outside oligonucleotide binding sites in the majority of successfully assayed SNPs.

We also found that the presence of secondary SNPs could be the cause of assay failure in two failed assays, namely AA_C431554_196 and AA_C444244_323. A SNP was detected by BWA/Samtools approach in the LSO binding site of AA_C431554_196 (Figure 4C). Based on the clustering profile of AA_C444244_323, all *A*. *mangium* genotypes failed to be called due to low signal intensities (Figure 4D), suggesting the presence of a secondary SNP in *A*. *mangium* sequence caused the failure of ASO carrying the allele A for *A*. *mangium* to bind to the SNP site. Since all *A*. *auriculiformis* samples were homozygous for B allele, all *Acacia* hybrids were heterozygous, carrying one allele from each parent (AA x BB → AB). The lower intensity of heterozygous cluster and compression towards the BB cluster is reflected by the presence of secondary SNP.

The flanking regions of the remaining three failed assays did not contain predicted exon-intron boundaries or secondary SNPs. However, we found that the genes from two failed SNP assays, namely AA_C431554_196 and AA_scaffold6698_331 shared 80-90% similarity with other contigs found in the transcriptomes. This suggested that assay failure might be associated with paralogous genes. In summary, we found no association between assay failure and design score. Half of the assay failures were associated with exon-intron boundaries while the rest might be caused by the presence of secondary SNPs and paralogous genes.

## 768 SNPs genotyping

Based on the findings in 96-plex validation and analysis of assay failure, we applied more stringent filtering in the SNP detection approach by increasing SNP score cutoff and removing genes with exon-intron boundaries and paralogous genes more than 90% similarity (Filter 3). Filtering of SNP score 100 and 150 resulted in 4,444 and 1,276 SNPs in AA6 and AM20, respectively. We selected these SNP score cutoffs to give a good tradeoff between reasonable polymorphism rate and number of SNPs as we would like to select SNPs that represent as many genes/contigs as possible. Out of 7,839 contigs in AArefseq, 2,859 contigs mapped to *M*. *truncatula* genes using blastn. Further 564 and 190 SNPs were removed from AA6 and AM20 SNPs dataset due to presence of predicted exon-intron boundaries within 35 bp upstream and downstream of SNP site. Contigs with predicted paralogous genes were removed before the final selection of SNPs. The final selection of 768 SNPs contained 566 contigs with 380 contigs (67.14%) mapped to *M*. *truncatula* genome. The mean SNP score for AA6 and AM20 excluding SNPs from 96-plex validation was 206 and 217, respectively.

In 768 SNPs genotyping (2 × 384-plex), 30 SNPs from both OPAs contained GenTrain score less than 0.25 but most were increased to more than 0.25 after manual adjustment. Out of 768 SNP assays, 710 assays (92.4%) were converted to working assays (Table 3). There were a total of 58 failed assays in which 23 SNPs (39.7%) had no hit to the *M*. *truncatula* genome. Out of 268 monomorphic SNPs, we observed about 117 interspecific SNPs (AA × BB → AB) and 78 SNPs displaying only AB genotypes. The clustering profiles of all interspecific SNPs except two experienced moderate to severe cluster compression. Although the success rate of 2 × 384-plex assay is higher than 96-plex assay, we found no significant difference between these two assays when tested using z-test (p≤0.05).

**Table 3:** Summary of 768 SNPs genotyping assay

**Population**	**WD and FL**	**WD**	**FL**
Assay failure	58	58	58
Monomorphic SNPs	269	284	565
Polymorphic SNPs	441	426	145
Total SNPs	768	768	768
Validation rate	62.10%	60%	NA
Conversion rate	57.40%	55.5%	18.9%

*NA = not available.

**Figure 5:**
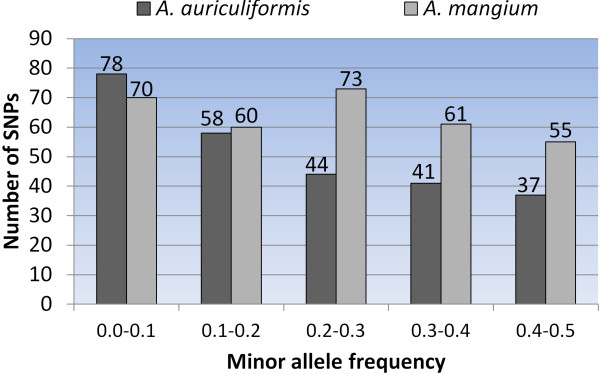
**Minor allele frequency of polymorphic SNPs in *Acacia auriculiformis *and *A.mangium *natural germplasms.**

An overall conversion rate of 57.5% was obtained for 768 SNPs genotyping. A total of 99.6% samples had an average call rate of at least 95%. The conversion rate for WD was 55.5% and lower conversion rate for FL was observed (18.9%), as expected. When the validated SNPs from 96-plex were excluded, the conversion rate was reduced to 56.2% and validation rate of 60.9% was obtained. Out of 426 polymorphic SNPs detected in WD population, 140 SNPs were polymorphic in FL population and thus, the SNPs transferability rate to other mapping population was 32.9%. In both 96 and 768 SNPs genotyping, we found a total of 258 and 319 polymorphic SNPs in the *A*. *auriculiformis* and *A*. *mangium* natural germplasms which represented 31.4% and 40.6%, respectively, out of the total SNPs (Table 4). SNPs with MAF of at least 0.10 represented 69.8% and 78.1% of the total polymorphic SNPs in the natural germplams (Figure 5).

**Table 4:** **SNPs transferability in *Acacia auriculiformis *and *A. mangium *natural germplasms**

**OPA**	***A*. *auriculiformis***	***A*. *mangium***
96-plex	31 (13*)	22 (15*)
aaOPA	166	104
amOPA	74	208
Total	258	319

*Polymorphic SNPs from 96-plex also present in 2 x 384-plex.

### Reproducibility of Illumina GoldenGate assay

**Figure 6:**
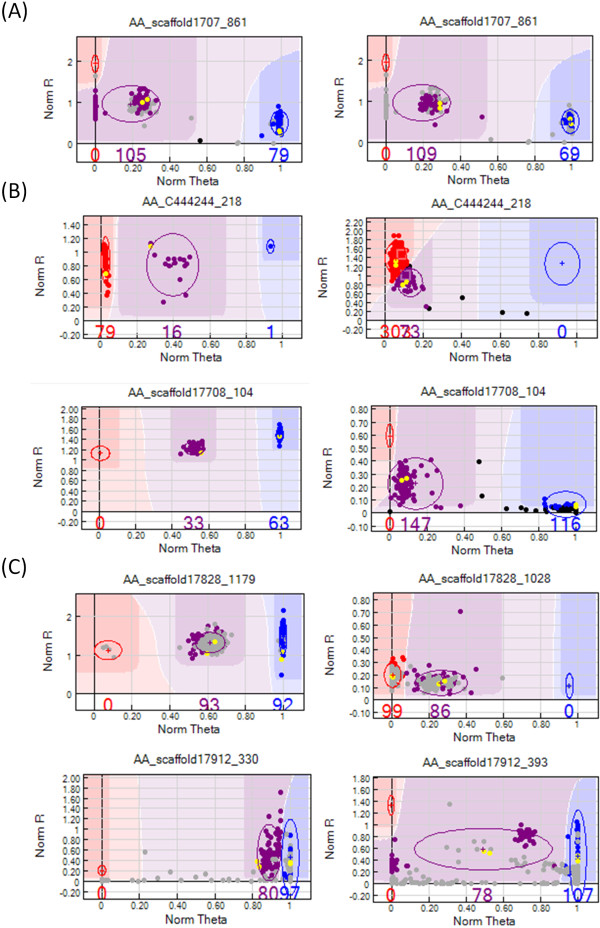
**Reproducibility of Illumina GoldenGate Assay clustering patterns.** (**A**) Clustering patterns of the same SNP for WD (left) and FL (right) population can be different as shown in AA_scaffold1707_861. Cluster located in 0.0 position along x-axis is identified as heterozygous cluster in WD population; (**B**) Clustering patterns of the same SNPs in 96-plex (left) and 384-plex (right) assays are not highly reproducible. For AA_C444244_218 (top), the homozygous and heterozygous clusters are indistinguishable in 384-plex genotyping compared to 96-plex genotyping. A shift in heterozygous cluster position and lower signal intensities was observed in AA_scaffold17708_104 (bottom); (**C**) Two different SNPs from the same gene based on WD population can exhibit different clustering patterns although under the same influence of paralogous genes. In AA_scaffold17828 (top), SNP at position 1179 (left) generated a good cluster profile, but SNP at position 1028 (right) showed cluster compression. For gene AA_scaffold17912 (bottom), severe cluster compression was observed for SNP at position 330 (left) while the cluster located at 0.0 along x-axis were identified as heterozygote based on Mendelian inheritance in SNP at position 393 (right). Replicates of the parents from WD population are highlighted in yellow; Excluded samples are masked in grey color; The data points in color represent genotype calls for each sample (red = AA; purple = AB; blue = BB; black = unknown/outlier). The x-axis (Norm Theta) represents angle of the center of cluster in normalized polar coordinate while y-axis (Norm R) represent normalized intensity. In theory, AA, AB and BB clusters should have normalized theta (x-axis) values of 0.0, 0.5 and 1.0, respectively. Otherwise, the clusters exhibit cluster compression.

We also assessed the reliability of Illumina GoldenGate assay by looking at the reproducibility of genotype calling. We found that the reproducibility of clustering profiles among two mapping populations was very high. Only 4 out of 130 polymorphic SNPs (3%) shared in both populations displayed different clustering profiles (Figure 6A). All four which were female markers had different positions of heterozygous cluster between populations.

Next, we examined the clustering patterns of the 29 SNPs genotyped in both 96-plex and 384-plex assays. Out of these, two SNPs initially thought to be polymorphic in 96-plex assay were identified as interspecific SNPs in 384-plex assay based on Mendelian inheritance and manual clustering. We found that the clustering patterns of 8 SNPs (27.6%) were not reproducible and additional manual clustering was required (See Additional file 1); 62% of the SNPs (18/29) suffered lower Norm R signal intensities in 384-plex compared to 96-plex assay. We observed that there were shifts of heterozygous clusters, which ranged from 0.1 to 0.4 along the x-axis. In SNP AA_C444244_218, the homozygous and heterozygous clusters were indistinguishable in 384-plex genotyping compared to 96-plex genotyping (Figure 6B). In another example, manual clustering based on Mendelian inheritance was used to identify the heterozygous cluster AA_scaffold17708_108 (Figure 6B).

We hypothesized that SNPs originating from the same gene are under the same influence of paralogous genes in the genome and thus, they should display the same clustering patterns based on three assumptions: 1) assay conditions are the same; 2) interaction with other assays are negligible; 3) different regions of the gene are influenced by paralogous genes in the same way. The only difference in these SNPs from the same gene was their flanking region sequence. To test our hypothesis, we compared the clustering profiles of 50 pairs of SNPs from the same gene from WD population (See Additional file 2). Out of 50 genes, five genes were removed from further analysis due to either sequence variation in flanking region or failed assay in either one ASO. Out of 45 examined genes, three genes did not display cluster compression. A total of 13 genes displayed the same degree of cluster compression in both SNPs. The remaining genes (64%) showed varying degree of cluster compression in both SNPs (Figure 6C). In addition, we observed several strange features in the clustering profile such as two sub-clusters within heterozygous cluster and heterozygous clusters located on 0.0 coordinate along x-axis (e.g. SNP AA_scaffold17912_393 in Figure 6C).

## Discussion

We have successfully developed high-throughput SNP assays for *A*. *auriculiformis* x *A*. *mangium* hybrids using Illumina GoldenGate Assay. The sensitivity of our *in silico* SNP detection approach was evaluated by comparison of validated SNPs detected using *in vitro* approach, selection of the most sensitive SNP detection tool and 96 SNPs validation. The assessment of assay failure has enabled us to identify exon-intron boundaries as the main cause and subsequently reduce this problem by using *M*. *truncatula* as reference genome. We further analyzed the clustering patterns and reproducibility of Illumina GoldenGate Assay to determine if the assay is suitable for a non-model species like *Acacia*. We have obtained SNP resources for linkage map construction, hybrid discrimination and genetic diversity studies of *A*. *auriculiformis* and *A*. *mangium*.

### *In silico* SNP detection

It is common to use more than one SNP prediction method in *in silico* SNP detection when using Next Generation Sequencing (e.g., GMAP and MAQ software were tested in soybean [16] and SOAP2 and Novoalign used in sorghum [38]). The comparison of *in silico* and *in vitro* SNP detection has led us to choose Bowtie over BWA as the default short read mapping tool. Bowtie has the advantage of providing fast mapping, allowing two mismatches and perform ungapped alignments. The high false negative rate observed in BWA/Samtools can be explained by lower number of mapped reads and thus fewer SNPs were detected. Our observation that Bowtie can map more reads and detect more SNPs compared to BWA is similar to another study [39] showing that Bowtie can map 25% more SNPs compared to MAQ, which is a replacement for BWA. The observation that most reads were mapped to the SNP site but the SNP was not called by samtools is probably due to low quality scores of the reads. The low sensitivity of BWA/Samtools method can cause the loss of valuable SNPs, which may affect the assay success rate. Since our primary objective is to develop SNPs for linkage mapping purpose, it is important to detect SNPs from a large set of genes that can be selected for good genome coverage.

The validation of 96 SNPs has provided important insights into the data quality of each dataset and sensitivity of our SNP detection approach. The observation that higher number of reads gives higher validation rate suggests that higher sequence coverage increases SNP accuracy. The good correlation between SNP score and validation rate showed that a higher SNP score stringency can be used to increase the validation rate. Initially, our study was designed to detect SNPs within both WD and FL populations. The poor sequence data of individual AM22 as indicated by the low validation rate makes it unsuitable for SNP detection. Due to the good SNP transferability rate to FL population, we decided to design high-throughput SNP assay from WD population. The improved SNP detection approach increased the assay conversion rate from 37.5% in 96-plex to 57.5% in 768 SNPs genotyping. The improved conversion rate is higher than the rate observed in European hake [23] and similar to other studies [40-42]. This rate is within the range of 60-70% observed in forest species (David Neale, personal communication). The conversion rate in *Acacia* is largely determined by the validation rate, as the assay success rate is very high.

The validation rate reported in this study is lower than other studies such as soybean [16], sorghum [38] and alfalfa [20] can be caused by several factors such as the use of *de novo* transcriptome assemblies as reference, mapping of single-end reads, low sequence coverage and presence of paralogous genes. We think that the presence of paralogous genes is the main contributor. Observation that 78 monomorphic SNPs exhibited AB genotype and almost all interspecific SNPs showed cluster compression in 768 SNPs genotyping suggests that 60% of monomorphic SNPs were also under the influence of paralogous genes. Paralogous genes can lead to false SNP detection by affecting fluorescence signal when the oligonucleotides were amplified in multiple locations with highly similar sequence in the genome. Paralogous genes are one big problem for *Acacia* because genome sequences are not available and the extent of genome duplication is unknown. If paralogous genes affect the detection of polymorphic SNPs, more stringent filtering will not improve validation rate. To overcome this problem in non-model species, some studies either use single copy genes (also known as Conserved Ortholog Set) [43,44] or remove genes that shared more than 90% sequence similarity [45]. Although our proposed methods were able to detect more paralogous genes by using a reference genome like *M*. *truncatula*, using transcriptome data alone is insufficient due to incomplete sequencing and failure to capture all paralogous genes in the genome. One possible solution is to identify paralogs within the transcriptome data using statistical methods such as QualitySNP [45]. Since all the samples were subjected to only one lane sequencing, low sequence coverage might be a problem as a redundancy of 13 reads were required to detect heterozygous SNPs [46]. With the improvement of sequence coverage, sequence quality and mapping algorithms, *in silico* SNP detection will become increasingly reliable in the future.

### Factors affecting assay failure in Illumina GoldenGate Assay

In this study, we obtained high assay success rate for *Acacia*, similar to both model and non-model species such as barley [47], soybean [16,48], pea [49], common bean [17], cassava [50], potato [51], tetraploid wheat [52] and *Eucalyptus grandis*[35]. The smaller genome size in *Acacia* may explain the higher assay success rate compared to other forest species [31,32,53]. According to Illumina, the likelihood of a SNP to be converted into a successful assay depends on the final SNP design score given by ADT and a minimum score of 0.6 is recommended. However, we found no correlation between design score and assay success rate. This observation is consistent with some studies [19,49] but contradictory to other studies [32,33]. Another study reported that there is no significant relationship between the design score and GenCall score, suggesting that the design score does not influence the quality of cluster separation [31]. Based on these results, we recommend using SNPs with design score of at least 0.4 for SNP genotyping in *Acacia*.

Our observation that half of the failed SNPs contained predicted exon-intron boundaries is similar to results from Anithakumari *et al*. study [51]. We predicted that only exon-intron boundaries located on oligonucleotide binding sites can cause assay failure. Our exon-intron boundary prediction is accurate considering that we were able to explain the difference between failed and successful assays containing exon-intron boundaries. Exon-intron boundary is a major concern for SNPs detected from ESTs or transcriptome data of non-model species. Since the flanking region is given in the gene coding region while genotyping were carried out using genomic DNA, the oligonucleotides will not be able to bind if an intron occurs within the flanking region. According to the Zhang *et al*. study [54], sequencing of EST amplicons revealed the presence of introns in 38% of the ESTs in oyster. To overcome the lack of genome sequence, many studies used reference genome from a related species (e.g., salmon genome for catfish [19], five fish genomes for European hake [23], chicken genome for mallard [18], Arabidopsis genome for carrot [55] and Solanaceae [56]). These studies utilized either a webserver [57] or custom scripts to predict the exon-intron boundaries. The limitation of exon-intron boundaries prediction using a reference genome is that its success depends on the conservation between the two genomes. Furthermore, exon-intron boundaries cannot be predicted for genes that are not found in the reference genome possibly due to gene loss and pseudogenes that occurred over the evolutionary history.

We found evidences that secondary SNPs may be responsible for a small proportion of failed assays. We predicted that presence of secondary SNPs can result in assay failure in two ways if a secondary SNP occurs in either one ASO: 1) In a testcross SNP, presence of secondary SNPs in the binding site of either one ASO will result in assay failure in half of the samples carrying that allele; 2) assay failure of all samples in the homozygote cluster of one allele and compression of heterozygous cluster toward homozygous cluster of the other allele. According to Tindall *et al*. study [58], the latter case can also occur due to presence of outliers and resulted in incorrectly genotyped samples. Presence of secondary SNP in flanking region can be a severe problem especially in the genotyping of highly heterozygous and outcrossing plants. However, the SNP frequencies in *A*. *auriculiformis* and *A*. *mangium*[12] are much lower compared to many plant species [34,35,59]. Therefore, presence of secondary SNPs in successfully assayed SNPs is not a major concern because all the genotypes can be easily differentiated and can be used for linkage mapping.

### Clustering and Reproducibility of Illumina GoldenGate Assay

To our knowledge, no other study has reported the reproducibility of clustering patterns although reproducibility of genotype calling was widely studied. Our observation that genotype calling is highly reproducible is consistent with results from another study [33]. However, high reproducibility of genotype calling does not necessarily mean high reproducibility of clustering patterns. Our results showed that the clustering profiles of almost one third of the SNPs shared between 96-plex and 384-plex assays are not reproducible, suggesting that manual clustering is needed for accurate genotype calling. The lower than expected reproducibility might be due to increased number of SNPs in the assay and interactions with other oligonucleotides within the assays. Our report that different clustering patterns observed in different mapping population was similar to Hyten *et al*. study [48]. Since reproducibility of clustering profiles between two mapping population is very high, manual clustering for each population is not required. Another important finding is the clustering patterns of SNPs from the same gene can vary greatly, suggesting that the flanking region of the SNP besides paralogous genes plays an important role in determining the clustering patterns. This finding supported the results from Wang *et al*. [19] who found that the quality of flanking region is very important.

Manual adjustment of clusters for custom Illumina GoldenGate Assay is often required for non-model species because the automated calling which was optimized for human genome can be unreliable. In addition, a feasible size of 96 samples is recommended for clustering to maximize statistical significance. GenCall software calls genotypes based on the assumption that there are three clusters in diploid organisms [28]. Even in verified human SNPs, Tindall *et al*. [58] provided evidence that GenCall software cannot cluster some SNPs accurately (e.g., SNP from very tight clusters and SNP that exhibited three clusters stacked vertically). Based on our experience, we found that the inclusion of samples from natural germplasm is very helpful in the identification of all three clusters especially when the SNPs were segregating in the mapping populations following testcross configuration. Most studies performed manual cluster adjustment using two common approaches, namely Hardy-Weinberg equilibrium for natural germplasms and Mendelian inheritance for mapping populations. Some other studies overcome this problem by using customized methods (e.g. manual adjustment as reported in polyploidy species [52], calculation of new cluster separation score in soybean [48] and other genotype calling software such as ALCHEMY in highly homozygous rice samples [60]). As manual inspection and definition of assay failure varies greatly among studies, assay success rates among studies are generally not comparable. Stringent manual clustering and calling assay failure will result in significant loss of marker information. Like other studies [58,61,62], we strongly recommend manual clustering for each SNP especially during initial SNP assay setup although it can be time-consuming for large number of SNPs. Since manual clustering is very subjective, several revisions of manual inspection may be required before the final clustering patterns can be decided.

### SNPs transferability to other populations

In this study, we found that the transferability of polymorphic SNPs detected in the parents of one mapping population to other mapping population and natural germplasms is similar to the study previously reported in grapevine [34]. Although the number of polymorphic SNPs in FL population is insufficient for a dense linkage map construction, these SNPs provide important markers to allow comparison and integration of linkage maps in the future. SNPs with MAF at least 0.10 is considered common and useful in the evaluation of natural germplasms. As the parents originated from natural populations, more SNPs are expected to be transferable to natural germplasms when screened with larger amount of samples. The polymorphic SNPs detected here should be viewed with caution because it is based on small sampling size and therefore, subjected to clustering errors. To study the genetic diversity of both species using these markers in the future, manual clustering must be performed carefully and evaluation based on Hardy-Weinberg equilibrium must be carried out.

## Conclusions

We have discovered large amount of SNP markers in the transcriptomes of *A*. *auriculiformis* and *A*. *mangium*. We have successfully developed high-throughput SNP genotyping assay in *A*. *auriculiformis* × *A*. *mangium* hybrids where most SNPs were converted to successful assays. Illumina GoldenGate SNP genotyping together with manual clustering can provide high quality genotypes for a non-model species like *Acacia*. The SNP genotyping assay has generated sufficient markers for linkage map construction of *Acacia* hybrid. The use of reference genome from the most closely related species will allow us to perform comparative genomics in the future. The identification of interspecific SNPs will be useful for genetic mapping of F_2_ or backcross populations, clone and hybrid discrimination. Good transferability rate to natural germplasms will allow genetic diversity assessment of *A*. *auriculiformis* and *A*. *mangium*.

## Materials and methods

### Plant materials

Plant materials were obtained from two *A*. *auriculiformis* × *A*. *mangium* F_1_ mapping populations, namely Wood Density (WD) and Fiber Length (FL) population. The WD and FL population were full sib crosses from AA6 × AM20 and AA3 × AM22, respectively. Samples from four parents were collected from Forest Research Institute Malaysia (FRIM), Kepong, while the progenies of both mapping populations were sampled from FRIM Station in Segamat, Johor and Borneo Tree Seed and Seedling Sdn. Bhd. in Bintulu, Sarawak. The natural germplasms of *A*. *auriculiformis* and *A*. *mangium* were sampled from the field plot in Universiti Kebangsaan Malaysia (UKM). These samples originated from ten populations of *A*. *auriculiformis* (SAR, MHR, MBN, JDG, DRV, ORV, GWC, BSW, MRV and PRV) and *A*. *mangium* (ERC, NSW, BAR, AVW, WMH, MHD, CRV, SRT, KRM and BNT) which were used in Sukganah 2011 study [63].

### Sample preparation for transcriptome sequencing

The sampling of plant tissues, namely young stem and inner bark tissues, and RNA extraction were carried out for AA3 and AM22 using the same method as described in the Wong *et al*. study [12]. Instead of using the pooled total RNA for transcriptome sequencing as in AA6 and AM20, first strand cDNA samples were synthesized from the total RNA samples of AA3 and AM22 using Clonetech SMART cDNA synthesis kit (Clonetech, USA) and second strand cDNA synthesis was carried out using Clonetech PCR kit (Clonetech, USA). The cDNA samples were normalized using Evrogen TRIMMER cDNA normalization kit (Evrogen, Russia) before sending to The GenePool (UK) for sequencing service. Paired-end sequencing was carried on one lane of flow cell on Illumina GAII for each sample. The short reads were filtered for SMART adaptors. The unfiltered sequences of AA3, AM22, AA6 and AM20 in FASTQ format are available in NCBI Sequence Read Achieve [SRA: SRR497265, SRR497266, SRR098315 and SRR098314].

### DNA extraction

Genomic DNA extraction of leaf tissues was carried out using Qiagen Tissuelyser II and DNeasy Plant mini kit. The quantity and quality of the DNA samples were assessed using Nanodrop ND-1000 Spectrophotometer (Thermo Scientific, USA) and Quant-i Picogreen assay (Invitrogen, USA) on Varioskan Flash Multimode Reader (Thermo Scientific, USA). The DNA samples were diluted to a final concentration of 20-50 ng/μl using TE buffer. The DNA samples were sent to International Rice Research Institute (IRRI) where SNP genotyping using Illumina GoldenGate Assay on Illumina BeadXpress machine was performed.

### SNP detection and Illumina GoldenGate Assay design

Reference sequences were selected from the *de novo* transcriptome assemblies of AA6 and AM20 (*de novo* transcriptome assembly methods described in [12]) due to better length and sequencing coverage. Each reference sequence must be present in both assemblies to ensure high sequence confidence in assay design of *Acacia* hybrid but only the contig with the longest length in either assembly was chosen. First, we used MUMMER Nucmer [64] to detect orthologous sequences between the two assemblies. Orthologous sequences with at least 200 bp and shared more than 90% similarity in nucleotide level were identified. The longest sequences were extracted using a Python script (See merge_scaffold.py in Additional file 3). The selected sequences were labelled with prefix “AA” or “AM” indicating what assemblies they originated from, followed by a “_” and suffix carrying the scaffold or contig number. The reference sequences were subsequently known as AArefseq (See Additional file 4).

The filtered reads from each parent were mapped separately to AArefseq using Bowtie-0.12.3 [65] and BWA-0.5.7 [66] with default setting in single end mode. SNPs from the alignments were called using Samtools-0.1.7 [67] and output in pileup format. Samtools assigned a SNP quality score (subsequently known as SNP score) to evaluate the reliability of SNP calling based on Phred-scaled probability that the consensus is identical to the reference. In this study, the allele from the reference sequence is known as reference allele while major allele is the most frequent allele present in the individual. The reference allele is not necessarily the major allele as the *de novo* assembler randomly chooses an allele when encounters a SNP or sequencing error. The frequency of reference allele, non-reference allele, minor allele and major allele were calculated and added to the pileup files using a Python script (See pileup2SNPcount.py in Additional file 3). Putative SNPs were extracted from the pileup files using the following parameters with AWK one-liner scripts: 1) Mapping and SNP score more than 20; 2) SNPs must be covered by at least 10 reads; 3) at least three non-reference alleles are present; 4) SNPs must be bi-allelic; 5) Minor Allele Frequency (MAF) must be at least 5%; 6) total frequency of major and minor allele must be at least 0.95. The total number of SNPs for all four individuals was calculated based on the total sum of SNPs from all four individuals minus the number of SNPs present in at least two individuals.

The FASTA sequences of AArefseq were converted to pileup format using a Python script (See fasta2pileup.py in Additional file 3). A consensus sequence for each parent in pileup format was produced using AWK one-liner scripts by: 1) Replacing reference sequence with filtered SNPs; 2) replacing consensus sequence with non-reference allele if reference allele was not present; 3) coding region with no read as N; 4) masking tri-allelic SNPs resulted from paralogous genes or sequencing errors as N; 5) correcting reference sequence for any sequencing error resulted from *de novo* transcriptome assembly. All the SNPs within both mapping populations were taken into consideration to allow cross amplification between populations. Therefore, the consensus sequence files from all four parents were combined into one file and a final consensus sequence was called using a Python script (See pileup2consensus_bwt.py in Additional file 3). Besides calling consensus, the script also masked all bi-, tri- and quad-allelic SNPs in the file with IUPAC code.

To design custom Illumina GoldenGate Assay, the flanking region of the SNP with a minimum of 50 bp upstream and 50 bp downstream sequence was required. The numbers of secondary SNPs, interspecific SNPs and Ns within the flanking region for each SNP were calculated from the final consensus sequences using a custom Python script (See pileup2countSNP_101.py in Additional file 3). We then extracted a minimum of 101 bp flanking region and removed any SNP with more than 4 ambiguous codes (including secondary SNPs, interspecific SNPs and Ns) in the flanking region. The SNPs were labelled with a prefix of the AArefseq sequence, followed by an “_” and suffix of SNP position within the scaffold or contig. The flanking sequences were saved in CSV format using a Python script (See pileup2seq.py in Additional file 3) and submitted to Assay Design Tool (ADT) [28]. Any SNP with final Illumina design score less than 0.4 were removed.

### Comparison of *in vitro* and *in silico* method

*In vitro* SNPs were identified from *cinammate 4hydroxylase* (C4H) and *cinnamyl alcohol dehydrogenase* (CAD) genes by sequencing 6 to 8 cDNA clones for each of the four parents [68]. These *in vitro* SNPs were validated using Illumina GoldenGate Assay in the natural germplasms [63]. The sequences of C4H and CAD genes from *de novo* transcriptome assemblies were aligned to full length *Acacia* hybrid cDNA sequence [Genbank: EU275980.1, EU275982.1] using NCBI Blastn Blast2sequences to identify shared regions. The flanking sequences of validated *in vitro* SNPs were aligned against the genomic sequence of C4H and CAD [Genbank: JN204274.1 and JN227538.1] and any SNP flanking region located outside the exonic region was removed. The 120 bp flanking sequences of the validated SNPs in these two genes [dbSNP: ss532671532, ss532671534, ss532671536, ss532671538, ss532671539, ss532671541, ss532671543, ss532671545, ss532671546, ss532671548] were aligned against the shared coding regions. All the SNPs located in the shared region were identified from the modified pileup file for each parent. The successfully genotyped, polymorphic SNPs and monomorphic SNPs from the validated SNPs were compared to the SNPs identified from *in vitro* and *in silico* approach. Based on these findings, the stringency of the SNP detection approach was increased by removing SNPs with SNP score less than 50 and MAF less than 8%.

## 96 SNPs validation

To evaluate the effect of SNP score on the SNP detection accuracy, a total of 96 SNPs with different SNP scores ranged from 50-228 were selected randomly (See Additional file 5). Of these, 12 SNPs shared by both *A*. *auriculiformis* parents (AA6 and AA3), 12 SNPs shared by both *A*. *mangium* parents (AM20 and AM22) and 18 SNPs for each parent were selected. A total of 30 SNPs were predicted to be polymorphic for each parent. Only testcross SNPs that were heterozygous in either one parent and segregating in the mapping population in ratio 1:1 were used in SNP genotyping. The SNPs validation was performed on 96 DNA samples using Illumina GoldenGate Assay on an Illumina BeadXpress platform according to manufacturer’s protocol. The samples consisted of four parents, 24 progenies for each mapping population, 21-22 individuals each for *A*. *auriculiformis* and *A*. *mangium* natural germplasms (Table 5). To assess the reproducibility of the genotyping assay, DNA samples from parent AM20 were duplicated.

**Table 5:** Plant materials used in SNPs validation and genotyping

	**Number of samples**	
	**96**-**plex**	**2 x 384**-**plex**
***Acacia *hybrid parents**		
AA6	1	2
AM20	2	2
AA3	1	2
AM22	1	2
**Natural germplasms**		
*Acacia auriculiformis*	22	10
*Acacia mangium*	21	11
**Mapping populations**		
AA6 x AM20 population	24	174
AA3 x AM22 population	24	181
Total number of Samples	96	384

Genotype calling was done using GenomeStudio Genotyping Module Version 1.8.4 with a minimum GenCall threshold of 0.25. The clustering of each SNP was adjusted manually by visual inspection. An assay was defined as failed assay if most of the intensities were lower than 0.10 and more than half of the genotypes from one mapping population cannot be called. A SNP was considered monomorphic if the parents shared the same homozygous genotype. An interspecific SNP that did not segregate in the mapping population because both parents were homozygous for one allele (AA × BB → AB) was considered a monomorphic SNP in this study. A SNP was polymorphic if at least one parent was heterozygous and the SNP was segregating in the mapping population following testcross (AB × AA → AB:AA = 1:1 or AB x BB → AB:BB = 1:1) or intercross (AB × AB → AA:AB:BB = 1:2:1) configuration. Mendelian inheritance in the mapping population and reproducibility within parent replicates were checked using Heritability and Reproducibility Error function.

The assay success rate was calculated based on the number of successful assays divided by total number of assays while the conversion rate was calculated based on number of polymorphic SNPs divided by total number of assays. Validation rate of the SNPs was calculated using the number of polymorphic SNPs divided by the number of successful assays. We also checked the significance and correlation between: 1) SNP design score and assay success rate; 2) validation rate and SNP score, using t-test and correlation test in Microsoft Excel. For comparison of SNPs detected from Bowtie and BWA alignments, the successfully validated SNPs were compared to putative SNPs detected by BWA. The validation rate was calculated based on the number of true SNPs also detected by BWA divided by the total number of SNPs also detected by BWA. The sensitivity of combined Bowtie and BWA approach was estimated by dividing the number of true SNPs detected from BWA alignments over the total number of true SNPs while the false negative rate is 1 minus sensitivity.

### Exon-intron boundary and paralogous genes prediction

Genome sequences, gene annotation and IMGAG files of *Medicago truncatula* version 3.5 (Release dated 25 August 2010) were downloaded from *M*. *truncatula* genome project website (http://www.jcvi.org/cgi-bin/medicago/download.cgi). For exon-intron boundary prediction, the start and stop position of each exon for each gene in *M*. *truncatula* genome was first extracted from IMGAG file and the length of each exon was calculated using an AWK script. The *Acacia* contigs were searched against *M*. *truncatula* full length transcripts using local NCBI Blast-2.2.25+ blastn algorithm (E-value ≤1E-30) and the results were output in both tab-delimited and XML formats. The XML Blastn results were simplified by extracting only useful columns with a custom Python script (See parsexml_biopython.py in Additional file 3). The best hit of *M*. *truncatula* full length transcripts for each *Acacia* contig based on the smallest E-value was extracted from both tab-delimited and simplified XML Blastn results.

A custom Python script (See findexon.py in Additional file 3) was designed to identify potential exon-intron boundaries in *Acacia* contigs by performing the following tasks: a) Obtain exon-intron boundary positions of the best hit *M*. *truncatula* transcript from extracted IMGAG file; b) predict the exon-intron boundary positions in *Acacia* contigs using alignments from extracted XML file; c) report SNPs that contain predicted exon-intron boundaries in 35 bp upstream and downstream of SNP site. The reported SNPs were removed from filtered SNP dataset of AA6 and AM20 using an AWK one-liner script. Known paralogous genes were identified by searching the contigs against other contigs in the transcriptomes using local NCBI Blast-2.2.25+ blastn algorithm (E-value ≤1E-30). Any SNP from a gene that shared more than 90% similarity in nucleotide level with another gene was removed. In addition, we also predicted paralogous genes by searching the contigs against the *M*. *truncatula* genes using the same method. Two or more contigs that map to the same *M*. *truncatula* genes were predicted as paralogous genes or fragmented contigs. Any contig that map to multiple *M*. *truncatula* gene transcripts are potential paralogous genes.

## 768 SNPs genotyping

**Figure 7:**
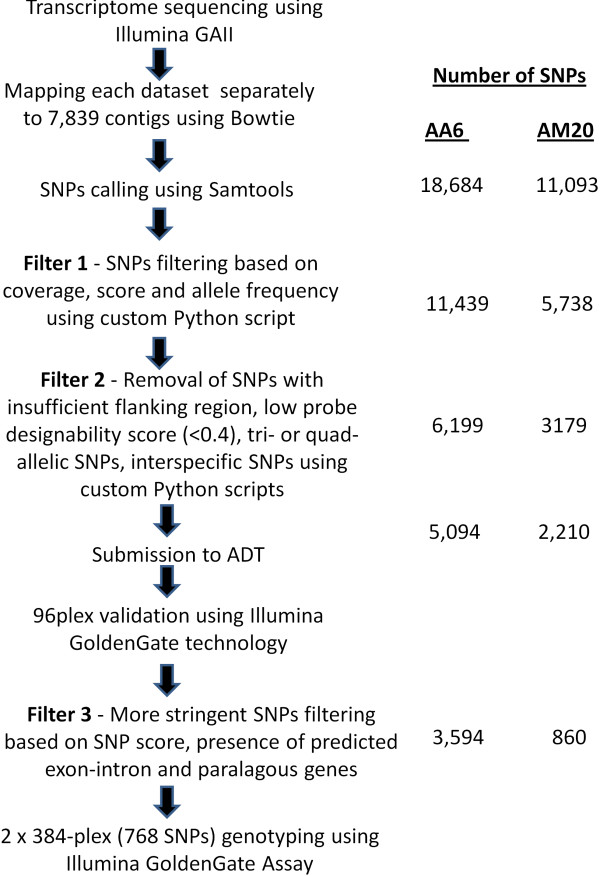
**Flowchart of SNP detection and assay design in *Acacia auriculiformis *x *A. mangium *hybrids.** Each step was briefly described on the left while the numbers on the right represent the number of SNPs after each filter. Only SNPs from individual AA6 and AM20 which were subsequently selected for 768 SNPs genotyping were shown.

Based on 96-plex validation results, more stringent filterings were applied to AA6 and AM20 SNP dataset using AWK script. 768 SNPs were selected for SNP genotyping as part of linkage map construction. The SNPs with predicted exon-intron boundaries and known or predicted paralogous genes (>;90% identity) were excluded. A flowchart summarizing the SNP detection approach for AA6 and AM20 is shown in Figure 7.

A set of 29 validated SNPs from 96-plex validation and 1 SNP from *caffeoyl CoA 3*-*O*-*methyltransferase* (CCoAOMT) gene were included. SNPs from CCoAOMT were detected using the same methods except the gene sequence of CCoAOMT1 [Genbank: JL053016] which was not included in AArefseq was used as reference. The SNPs were designed in two 384-plex Oligonucleotide Pooled Assays (OPAs), namely aaOPA and amOPA. Any two SNPs from the same gene or located less than 50 bp away were placed in separate OPA. All the SNPs in amOPA were markers from AM20 while aaOPA contained mainly SNPs from AA6 and a small proportion SNPs from AM20 (See Additional file 5).

The SNPs genotyping was carried out on 384 DNA samples (Table 5) using Illumina GoldenGate Assay on Illumina BeadXpress machine. Each parent was genotyped in duplicates and each replicate was placed in separate 96-well plates to detect interplate variation. Genotype calling was carried out as described for 96-plex genotyping. Outliers were identified when call rate was plotted against p10 GC. After excluding outliers, the number of samples with at least 95% call rate was calculated based on the total number of polymorphic SNPs. The separation patterns or clusters of each SNP were inspected separately for each mapping population and natural germplasm. A SNP was considered polymorphic in the natural germplasm if at least one out of six individuals carries a different genotype. MAF for each SNP of each natural germplasm was calculated based on the number of minor alleles divided by total number of alleles.

## Competing interests

The authors declared that they have no competing interests.

## Authors’ contributions

MW prepared the samples, designed the experiment, analyzed the data and drafted the manuscript. WR and CC conceived the study, participated in the design and coordination of the study. All the authors read and approved the final manuscript.

## Supplementary Material

Additional file 1 **Clustering profiles of 29 SNPs in 96-plex and 384-plex genotyping.** This Word document file contains a table showing the clustering profiles of 29 SNPs. The clustering profiles in 96-plex and 384-plex genotyping are shown side-by-side to indicate the reproducibility of Illumina GoldenGate Assay. Click here for file

Additional file 2 **Clustering profiles of SNPs from 50 genes in 768 SNPs genotyping.** This Word document file contains a table showing the clustering profiles of 50 genes. The clustering profiles of the two SNPs from the same gene are shown side-by-side. Click here for file

Additional file 3 **Python scripts used in SNP detection.** This compressed file in tar.gz format contains six Python scripts used in SNP detection. The title of each script is the same as the title that appeared in the text. Click here for file

Additional file 4 **Reference sequences used in SNP detection.** This compressed file in tar.gz format contains a FASTA file of AArefseq (AArefseq_merge.fa). Click here for file

Additional file 5 **A SNP summary of 96 SNPs validation and 768 SNPs genotyping.** This Excel file contains three sheets, namely “96plex”, “aaOPA3” and “amOPA3”. Each sheet contains SNP information of dbSNP id, SNP contig and position, design score, SNP score, homologous genes in Mt3.5, assay success, polymorphism, mapping population, minor allele frequency in *A*. *auriculiformis* and *A*. *mangium* natural germplasms. Description of each column can be found on the right in sheet “96plex”. Click here for file
