# Supplementary material for: Development of high-throughput SNP-based genotyping in Acacia auriculiformis x A. mangium hybrids using short-read transcriptome data
Source: BMC Genomics. 2012 Dec 24;13:726. doi: 10.1186/1471-2164-13-726 (PMC3556151; doi:10.1186/1471-2164-13-726)
Supplement: Additional file 1 — Clustering profiles of 29 SNPs in 96-plex and 384-plex genotyping. This Word document file contains a table showing the clustering profiles of 29 SNPs. The clustering profiles in 96-plex and 384-plex genotyping are shown side-by-side to indicate the reproducibility of Illumina GoldenGate Assay. [file 1471-2164-13-726-S1.docx]

Additional File 4. Clustering profiles of 29 SNPs indicating the reproducibility of clustering patterns between 96plex and 384plex.

| No | 96plex | 384plex | Reproducible | Low intensity |
| --- | --- | --- | --- | --- |
| 1 | 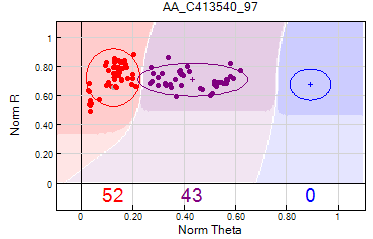 | 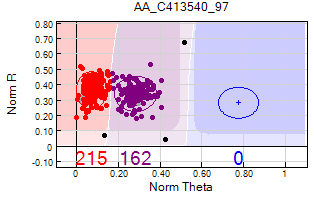 | No | Yes |
| 2 | 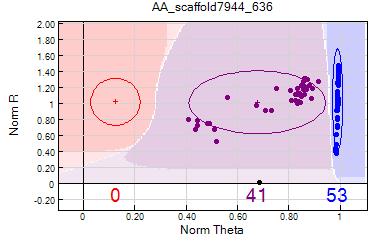 | 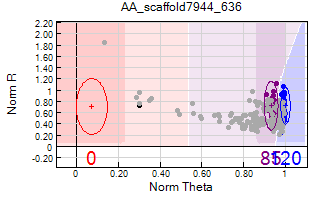 | No | Yes |
| 3 | 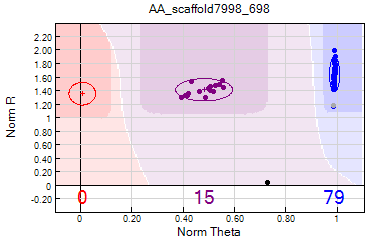 | 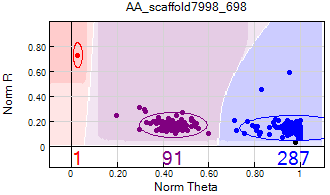 | Yes | Yes |
| 4 | 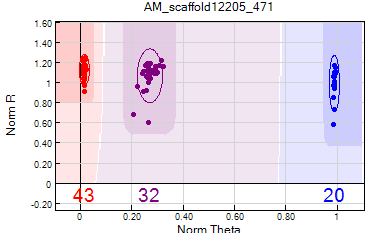 | 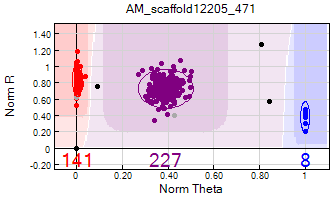 | Yes | Yes |
| 5 | 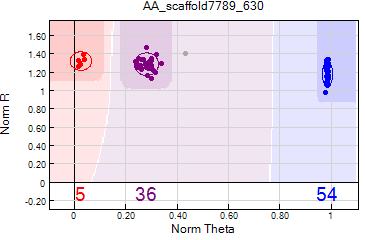 | 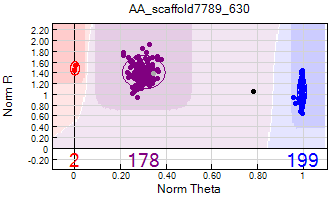 | Yes | No |
| 6 | 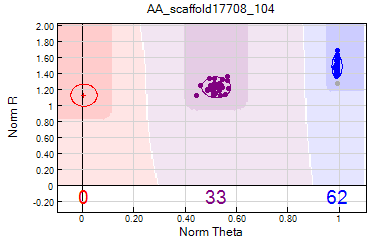 | 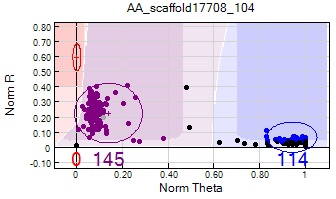 | No | Yes |
| 7 | 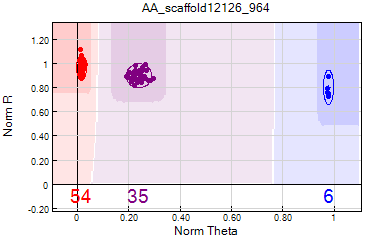 | 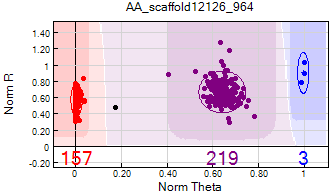 | No | Yes |
| 8 | 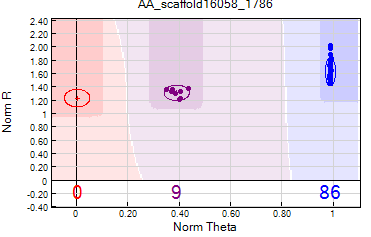 | 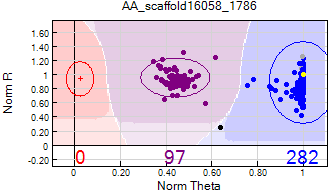 | Yes | Yes |
| 9 | 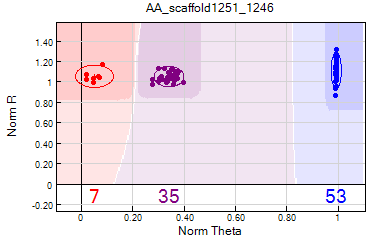 | 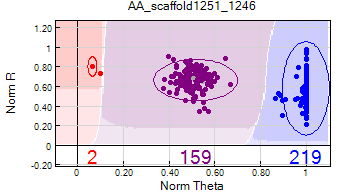 | Yes | Yes |
| 10 | 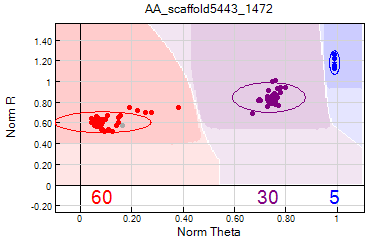 | 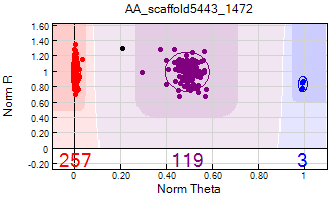 | Yes | No |
| 11 | 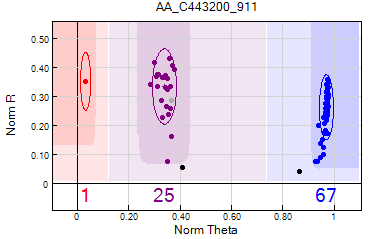 | 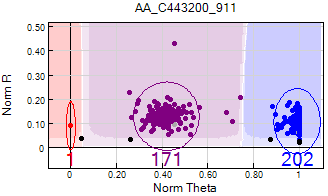 | Yes | Yes |
| 12 | 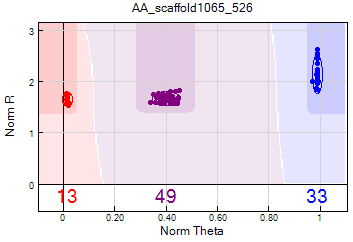 | 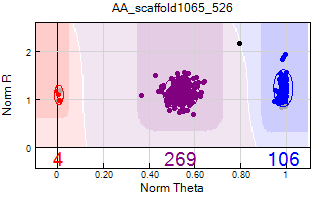 | Yes | Yes |
| 13 | 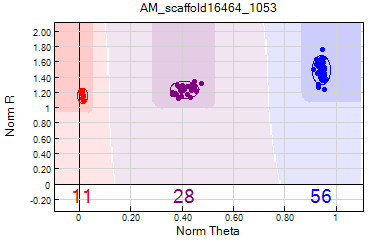 | 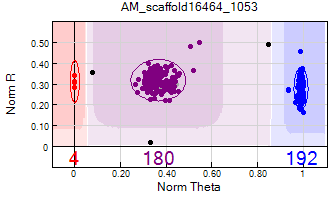 | Yes | Yes |
| 14 | 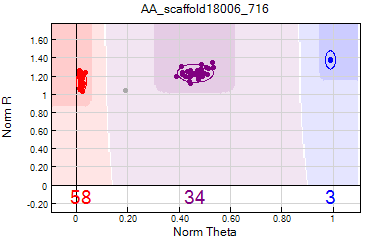 | 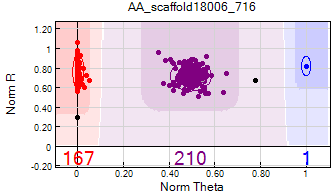 | Yes | Yes |
| 15 | 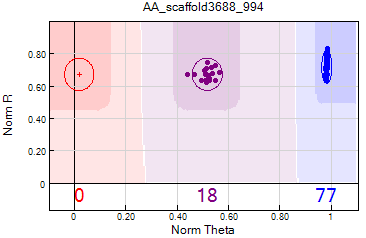 | 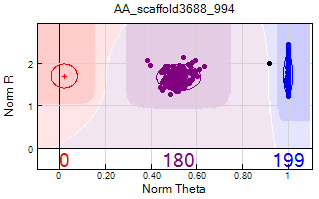 | Yes | No |
| 16 | 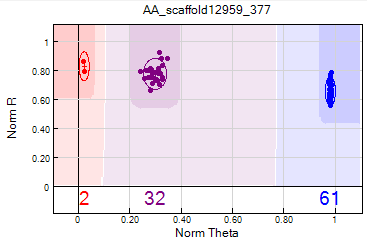 | 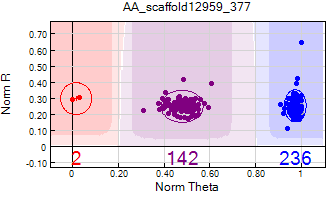 | Yes | Yes |
| 17 | 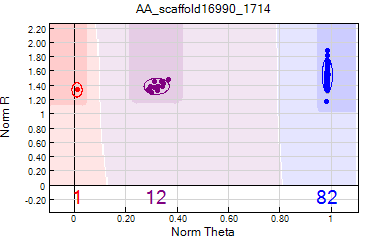 | 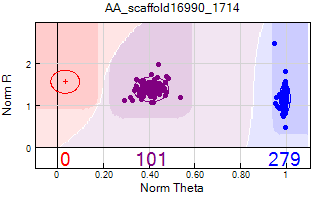 | Yes | No |
| 18 | 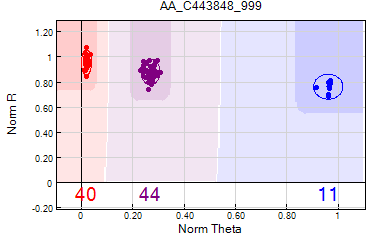 | 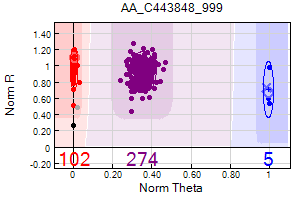 | Yes | No |
| 19 | 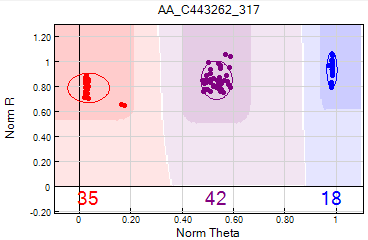 | 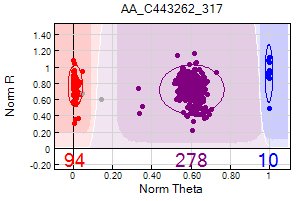 | Yes | No |
| 20 | 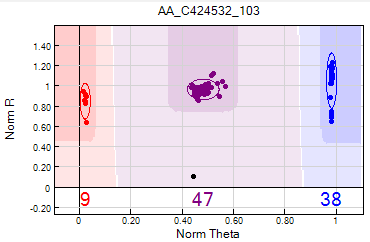 | 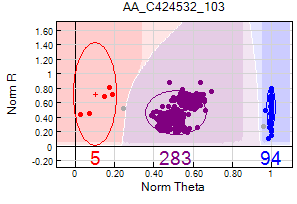 | Yes | Yes |
| 21 | 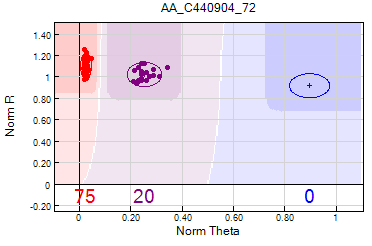 | 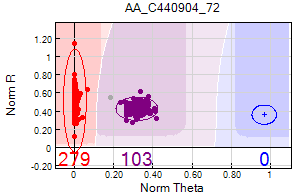 | Yes | Yes |
| 22 | 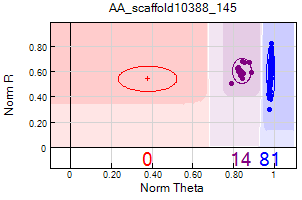 | 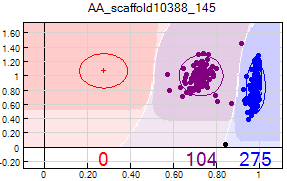 | Yes | No |
| 23 | 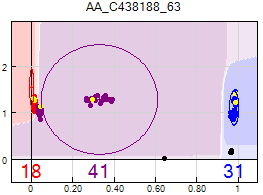 | 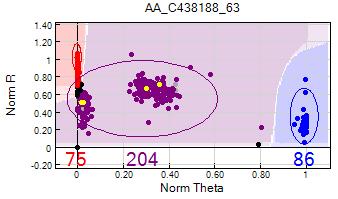 | Yes | Yes |
| 24 | 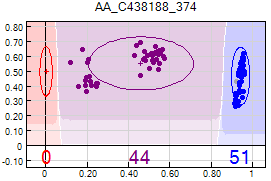 24 | 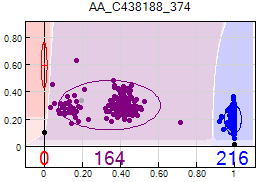 | No | Yes |
| 25 | 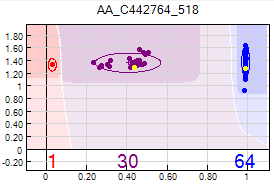26 | 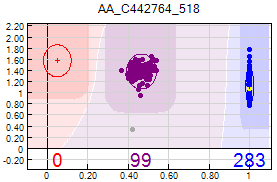 | Yes | No |
| 26 | 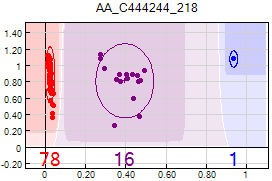  AA_C444244_218 (poly in 96plex, Mono in 384plex) | 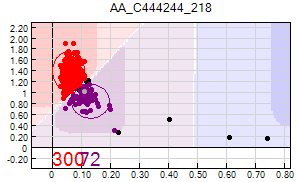 | No | No |
| 27 | 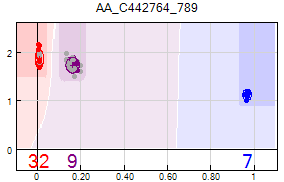 | 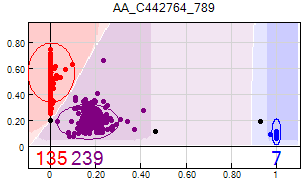 | No | Yes |
| 28  (Mono) | AA_C444244_323 (Mono)  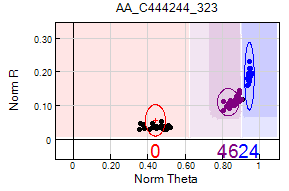 | 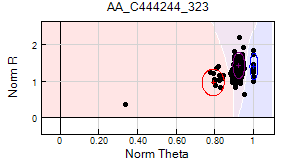 | No  (monomorphic) | No |
| 29  (Mono) | 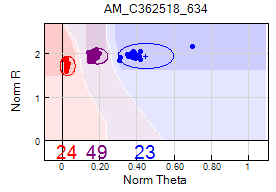 | 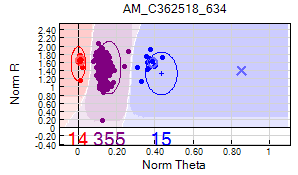 | Yes (monomorphic) | No |
