# Supplementary material for: Development of high-throughput SNP-based genotyping in Acacia auriculiformis x A. mangium hybrids using short-read transcriptome data
Source: BMC Genomics. 2012 Dec 24;13:726. doi: 10.1186/1471-2164-13-726 (PMC3556151; doi:10.1186/1471-2164-13-726)
Supplement: Additional file 2 — Clustering profiles of SNPs from 50 genes in 768 SNPs genotyping. This Word document file contains a table showing the clustering profiles of 50 genes. The clustering profiles of the two SNPs from the same gene are shown side-by-side. [file 1471-2164-13-726-S2.docx]

| No | Male SNP | Female SNP | Compression | Degree of compression between 2 SNPs |
| --- | --- | --- | --- | --- |
| 1 | 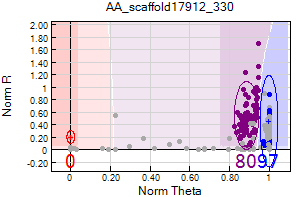 | 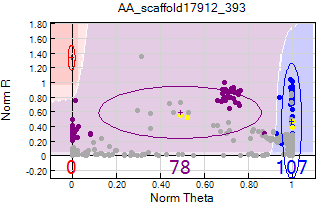 | Yes | Different |
| 2 | 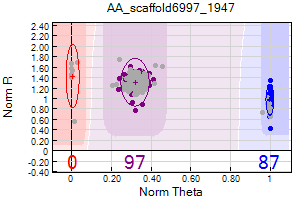 | 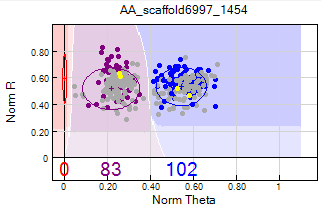 | Yes | Different |
| 3 | 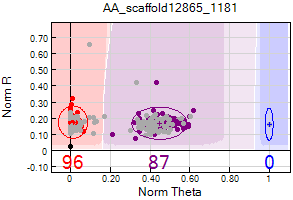 | 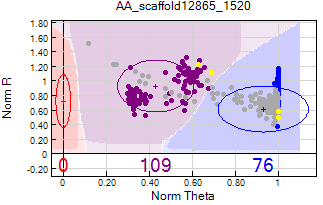 | Yes | Slight |
| 4 | 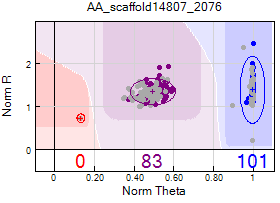 | 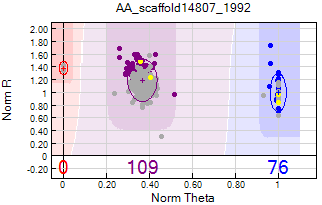 | Yes | Slight |
| 5 | 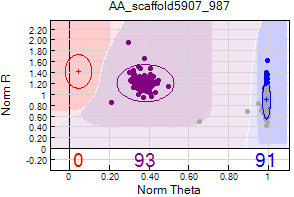 | 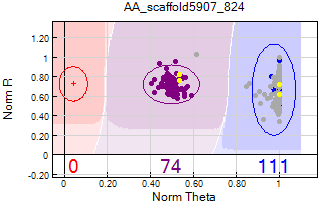 | Yes | Slight |
| 6 | 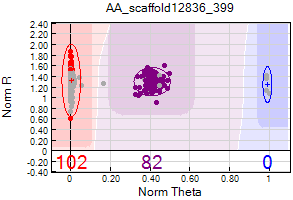 | 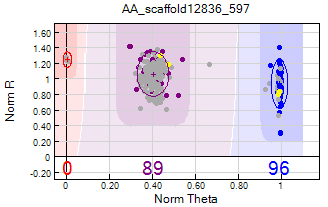 | Yes | Slight |
| 7 | 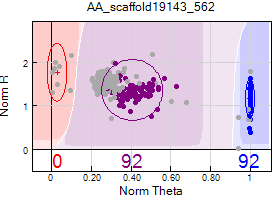 | 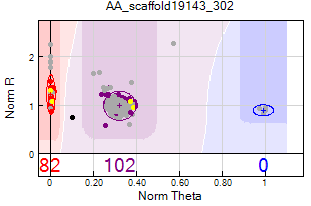 | Yes | Different |
| 8 | 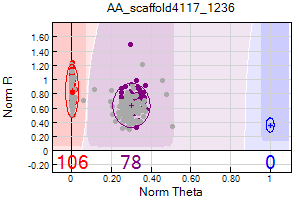 | 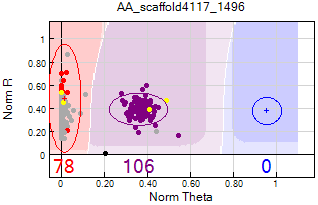 | Yes | Different |
| 9 | 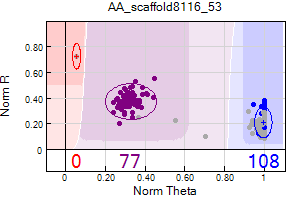 | 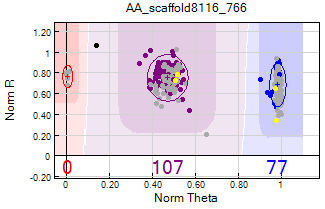 | Yes | Different |
| 10 | 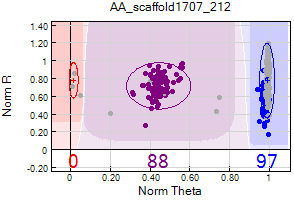 | 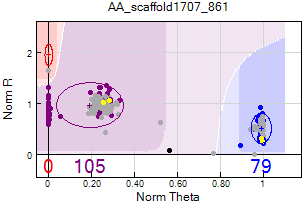 | Yes | Different |
| 11 | 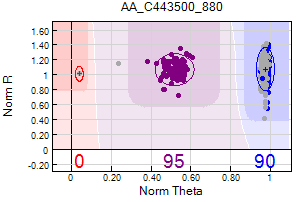 | 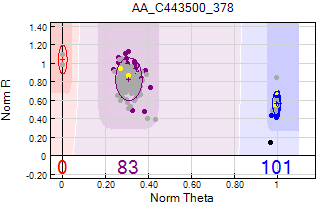 | Yes | Different |
| 12 | 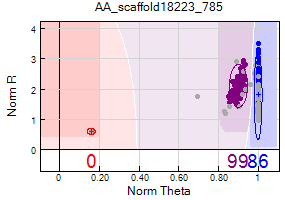 | 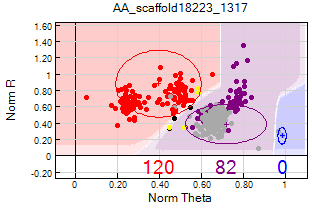 | Yes | Different |
| 13 | 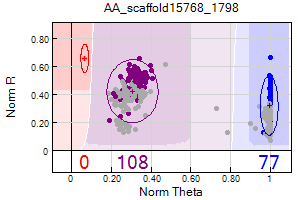 | 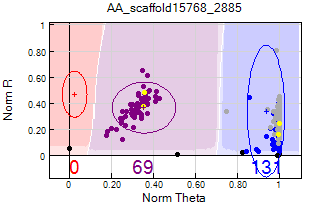 | Yes | Slight |
| 14 | 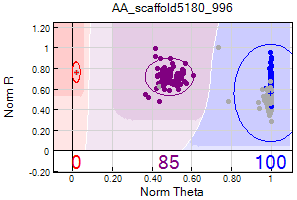 | 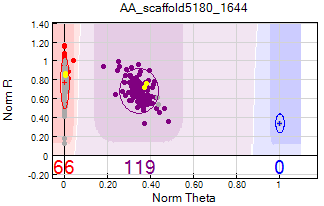 | Yes | Different |
| 15 | 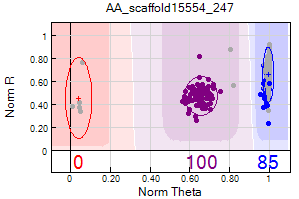 | 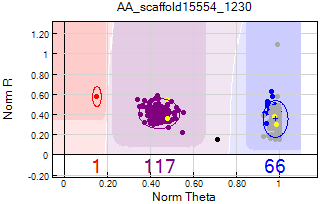 | Yes | Different |
| 16 | 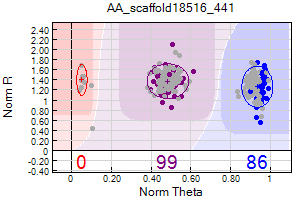 | 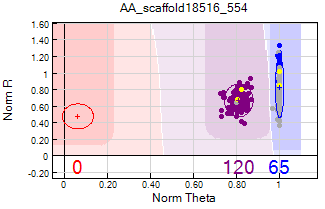 | Yes | Different |
| 17 | 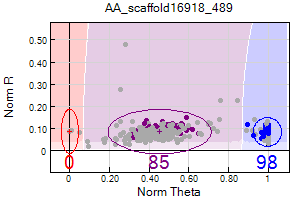 | 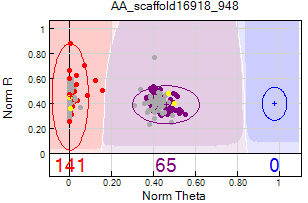 | No | - |
| 18 | 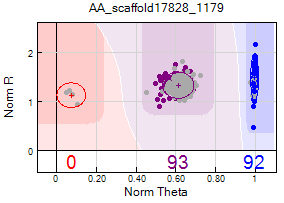 | 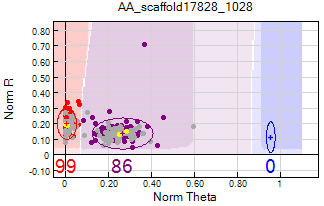 | Yes | Different |
| 19 | 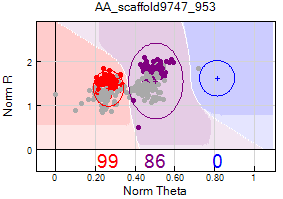 | 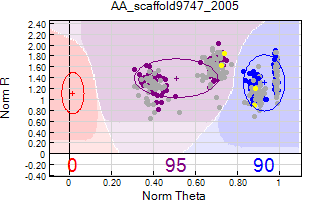 | Yes | Different |
| 20 | 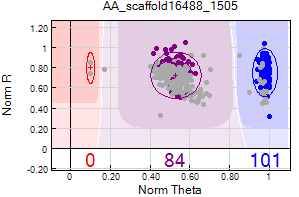 | 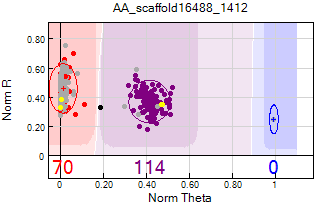 | Yes | Different |
| 21 | 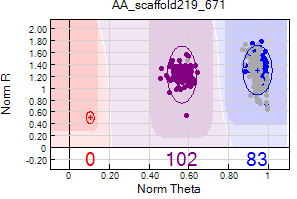 | 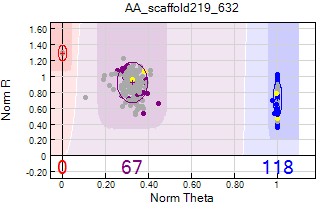 | Yes | Different |
| 22 | 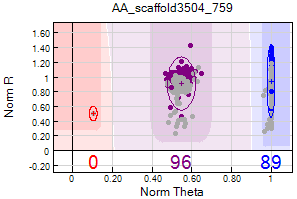 | 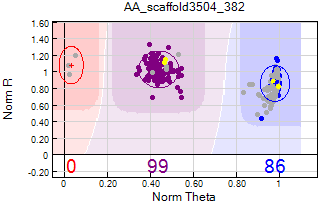 | Yes | Slight |
| 23 | 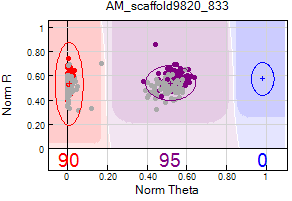 | 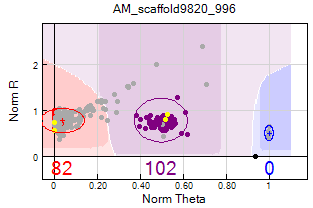 | No | - |
| 24 | 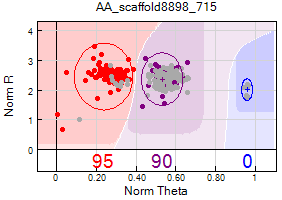 | 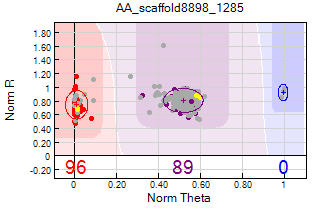 | Yes | Different |
| 25 | 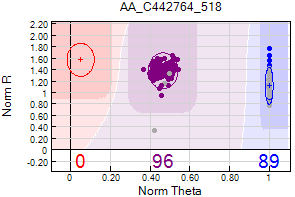 | 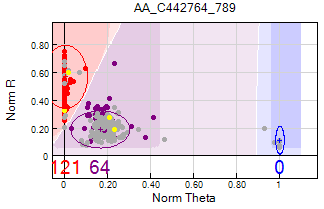 | Yes | Different |
| 26 | 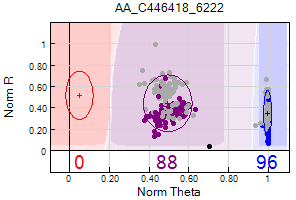 | 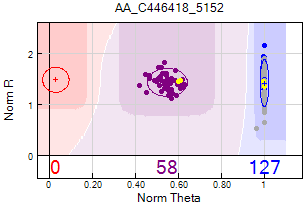 | Yes | Different |
| 27 | 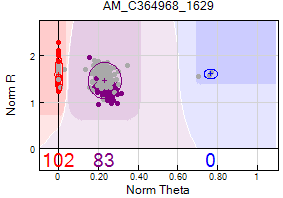 | 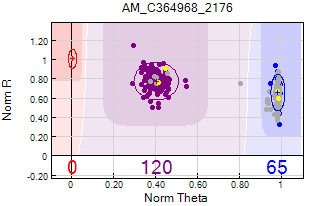 | Yes | Different |
| 28 | 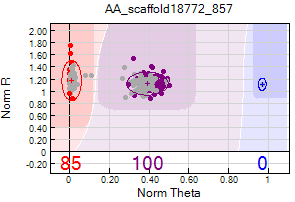 | 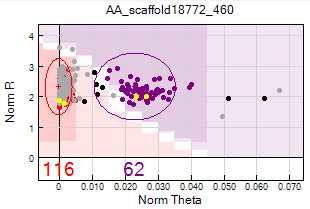 | Yes | Different |
| 29 | 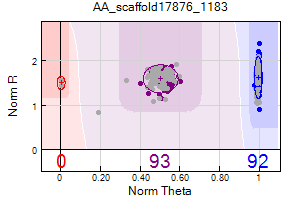 | 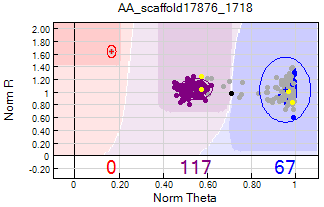 | No | - |
|  |  |  |  |  |
| 30 | 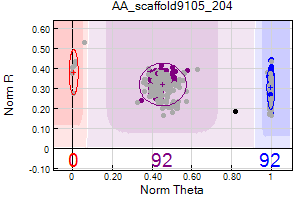 | 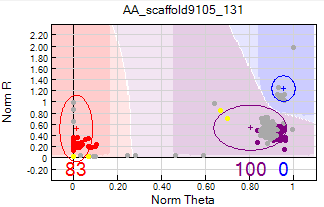 | Yes | Different |
| 31 | 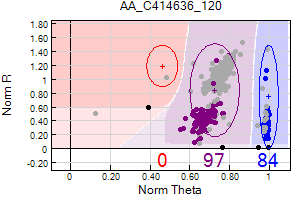 | 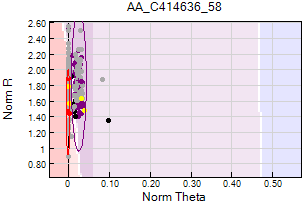 | Yes | Different |
| 32 | 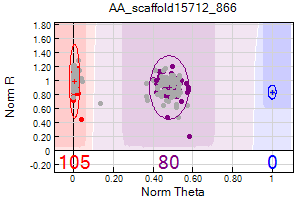 | 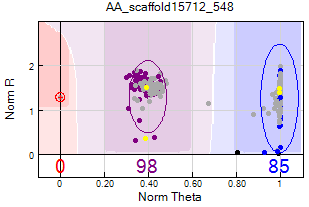 | Yes | Slight |
| 33 | 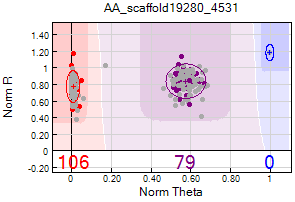 | 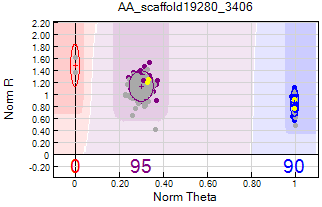 | Yes | Different |
| 34 | 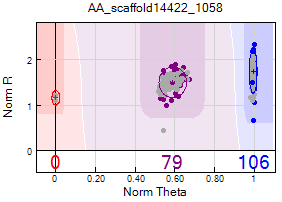 | 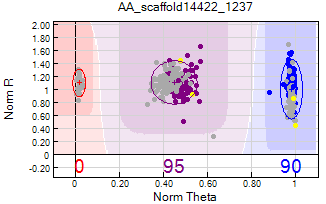 | Yes | Slight |
| 35 | 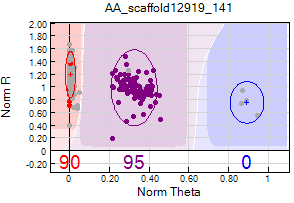 | 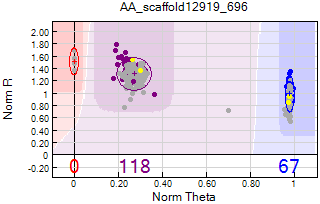 | Yes | Different |
| 36 | 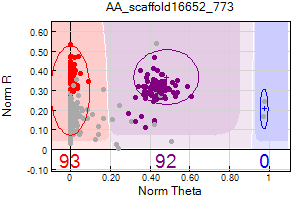 | 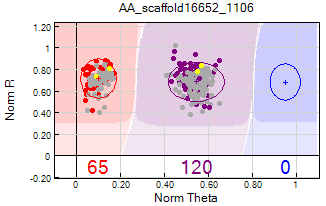 | Yes | Slight |
| 37 | 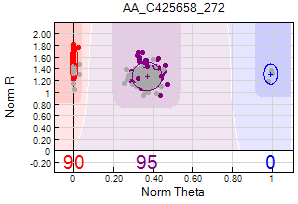 | 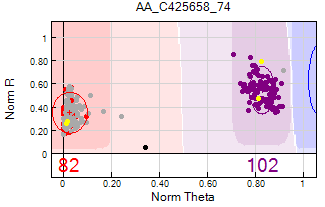 | Yes | Different |
| 38 | 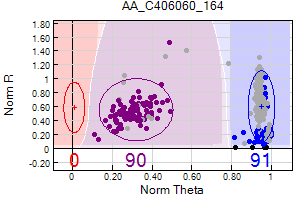 | 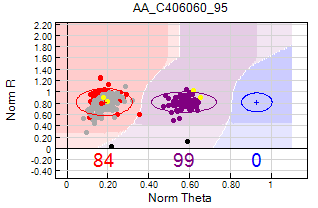 | Yes | Different |
| 39 | 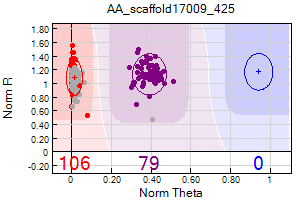 | 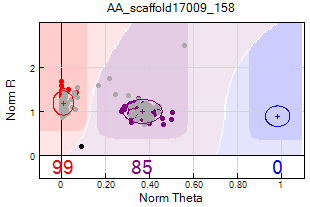 | Yes | Slight |
| 40 | 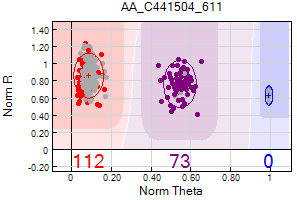 | 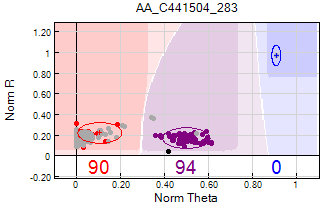 | Yes | Slight |
| 41 | 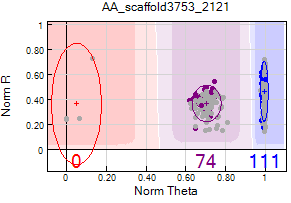 | 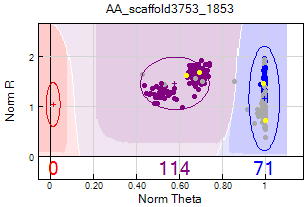 | Yes | Different |
| 42 | 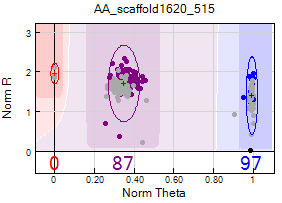 | 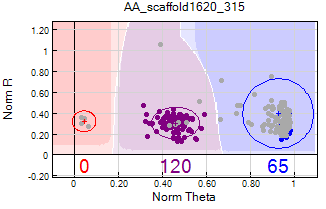 | Yes | Different |
| 43 | 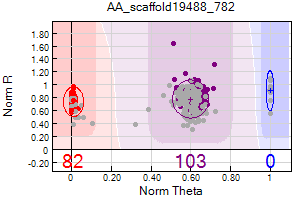 | 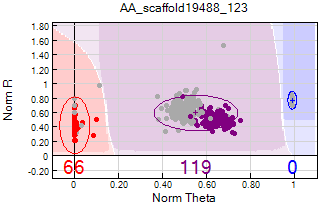 | Yes | Slight |
| 44 | 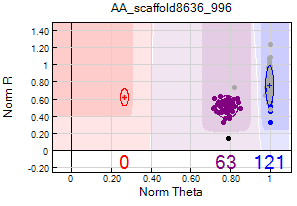 | 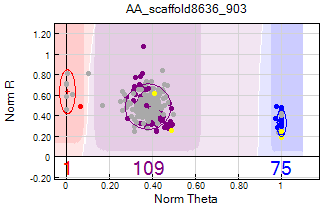 | Yes | Different |
| 45 | 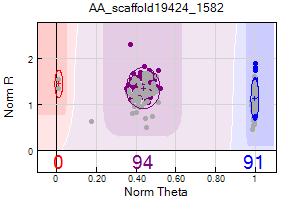 | 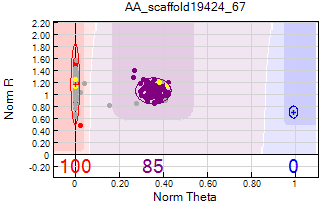 | Yes | Slight |

SNPs that were removed from further analysis (exhibited cluster compression due to flanking region or assay failure)

| No | Male SNP | Female SNP |
| --- | --- | --- |
| 1 | 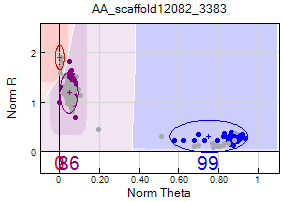 | 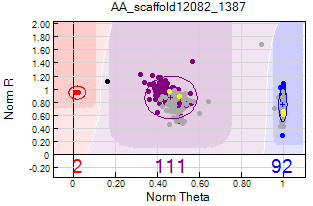 |
| 2 | 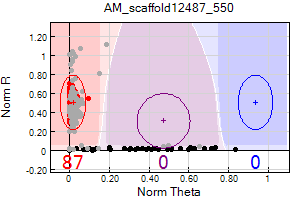 | 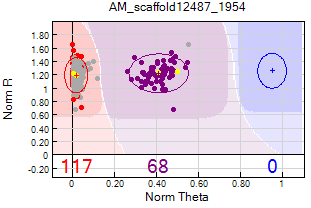 |
| 3 | 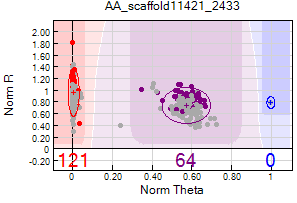 | 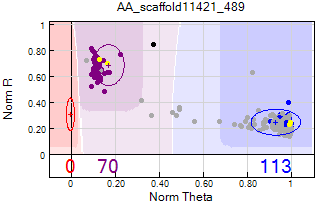 |
| 4 | 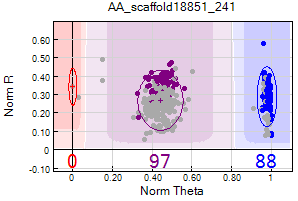 | 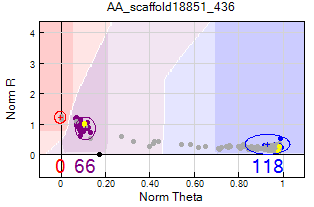 |
| 5 | 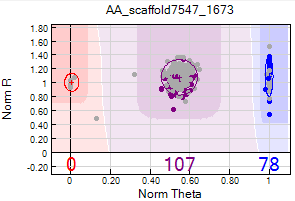 | 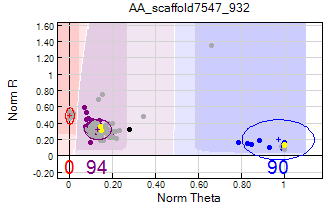 |
